# Supplementary material for: Bayesian analysis of isothermal titration calorimetry for binding thermodynamics
Source: PLoS One. 2018 Sep 13;13(9):e0203224. doi: 10.1371/journal.pone.0203224 (PMC6136728; doi:10.1371/journal.pone.0203224)
Supplement: S1 Appendix — (PDF) [file pone.0203224.s001.pdf]

Simple two-component (1:1) association binding model

In a simple two-component (1:1) complexation reaction, we have reversible association between a ligand L and a receptor R

$$R + L \overset{K_a}{\rightleftharpoons} RL, \tag{S1}$$

where the *association constant* K_a or the binding free energy ΔG is related to concentrations $[X]$ of the microscopic species at equilibrium by,

$$K_a \equiv \exp(-\beta \Delta G) = \frac{[RL]}{[R][L]}. \tag{S2}$$

With each injection, three effects will contribute to the true quantity of heat q_i^* liberated due to injection i: (1) the association of R with L, (2) the dilution of ligand and buffer into the protein solution (as most solutions are nonideal), and (3) the mechanical heat produced by the injection and stirring. We subsume the latter two components into a single term ΔH_0, and write

$$q_i^* = \Delta H * V_0 * \left([RL]_i - d_i * [RL]_{i-1}\right) + \Delta H_0, \tag{S3}$$

where ΔH is the enthalpy change associated with binding, $[RL]_i$ is the complex equilibrium concentration after injection i, V_0 is the cell volume, and d_i is the dilution factor after an injection with volume v_i, defined as

$$d_i = 1 - (v_i/V_0). \tag{S4}$$

In what follows, we will express the complex equilibrium concentration after injection i, $[RL]_i$, in terms of K_a, the cell volume V_0, the initial concentration of the receptor $[R]_0$, and syringe concentration of the ligand $[L]_s$.

The total quantity (number of moles) of receptor R_i and ligand L_i in the cell after injection i is given by

$$R_i \quad = \quad V_0\,[R]_0 * d_{\text{cum},i}, \tag{S5}$$

$$L_i \quad = \quad V_0 * [L]_s * (1 - d_{\text{cum},i}), \tag{S6}$$

where $d_{\text{cum},i}$ is the cumulative dilution factor given by

$$d_{\text{cum},i} = \prod_i 1 - (v_i/V_0). \tag{S7}$$

This model accounts for perfusion of receptor and ligand from the cell at a constant cell volume while assuming the mixing after injection is instantaneous as in [**?**].

Conservation of mass gives us the constraints

$$R_i = V_0\,([R]_i + [RL]_i) \tag{S8}$$

$$L_i = V_0\,([L]_i + [RL]_i). \tag{S9}$$

Combining Eqs. S2 and S9 gives

$$[R]_i = \frac{[RL]_i}{K_a \frac{L_i}{V_0} - K_a [RL]_i}. \tag{S10}$$

Substituting Eq. S10 into Eq. S8 yields a quadratic equation in the complex equilibrium concentration $[RL]_i$:

$$[RL]_i^2 - \left(\frac{R_i}{V_0} + \frac{1}{K_a} + \frac{L_i}{V_0}\right)[RL]_i + \frac{R_i \cdot L_i}{V_0^2} = 0, \tag{S11}$$

where the only solution that satisfies $0 \leq [RL]_i \leq \min\{[R], [L]_i\}$ is

$$[RL]_i = \frac{1}{2V_0}\left\{\left(R_i + L_i + \frac{V_0}{K_a}\right) - \left[\left(R_i + L_i + \frac{V_0}{K_a}\right)^2 - 4R_iL_i\right]^{\frac{1}{2}}\right\}. \tag{S12}$$
