# Supplementary material for: Bayesian analysis of isothermal titration calorimetry for binding thermodynamics
Source: PLoS One. 2018 Sep 13;13(9):e0203224. doi: 10.1371/journal.pone.0203224 (PMC6136728; doi:10.1371/journal.pone.0203224)
Supplement: S1 Table — (PDF) [file pone.0203224.s002.pdf]

Measurement No.	Initial injection (μL)	Volume of main injections (μL)	Amount of main injections (excluding initial inject.)	Initial delay (sec)	Injection spacing (sec)	Differential power (μcal s^{-1})
1	0.3	1.5	26	180	180	5
2	0.3	1.5	26	180	180	5
3	0.3	1.5	26	180	180	5.35
4	0.3	1.3	26	180	180	5.4
5	0.3	1.5	26	180	180	5
6	0.3	1.5	26	180	180	5
7	0.3	1.5	24	180	180	5
8	0.5	1.2	24	180	180	5
9 [a]	0.3	1.3	24	180	170	5
10 [a]	0.3	1.3	19	180	170	5

[a] published in Krimmer et al. [1]

Table A. Experimental parameters of ligand 1:thermolysin ITC measurements.

Measurement No.	Initial injection (μL)	Volume of main injections (μL)	Amount of main injections (excluding initial inject.)	Initial delay (sec)	Injection spacing (sec)	Differential power (μcal s^{-1})
1	0.3	1.3	26	180	180	5.4
2	0.3	1.3	24	180	170	5
3	0.3	1.5	26	180	180	5.4
4	0.3	1.3	24	180	170	5
5	0.3	1.5	26	180	180	5.6
6	0.3	1.5	26	180	180	5
7	0.3	1.3	24	180	170	5
8	0.3	1.3	24	180	170	5
9 [a]	0.3	1.3	19	180	170	5
10 [a]	0.3	1.3	19	180	170	5
11 [a]	0.3	1.3	19	180	170	5

[a] published in Krimmer et al. [1]

Table B. Experimental parameters of ligand 2:thermolysin ITC measurements.

Measurement No.	Initial injection (μL)	Volume of main injections (μL)	Amount of main injections (excluding initial inject.)	Initial delay (sec)	Injection spacing (sec)	Differential power (μcal s^{-1})
1	0.3	1.5	24	180	170	5
2	0.3	1.5	24	180	180	5.6
3	0.3	1.5	26	180	180	5.6
4 [a]	0.3	1.5	19	180	170	5
5 [a]	0.3	1.5	24	180	180	5.6
6 [a]	0.3	1.5	19	180	170	5
7	0.3	1.5	24	180	180	5.6
8 [a]	0.3	1.5	19	170	180	5
9	0.3	1.5	26	180	180	5.6

[a] published in Krimmer et al. [1]

Table C. Experimental parameters of ligand 3:thermolysin ITC measurements.

References

[1] Krimmer, S. G., Betz, M., Heine, A., and Klebe, G. (2014). Methyl, ethyl, propyl, butyl: futile but not for water, as the correlation of structure and thermodynamic signature shows in a congeneric series of thermolysin inhibitors. *ChemMedChem*, 9(4):833–846.
