# Supplementary figures and images for: Bayesian analysis of isothermal titration calorimetry for binding thermodynamics
Source: PLoS One. 2018 Sep 13;13(9):e0203224. doi: 10.1371/journal.pone.0203224 (PMC6136728; doi:10.1371/journal.pone.0203224)

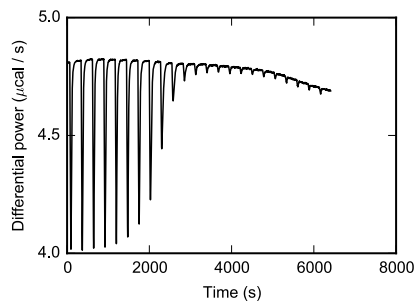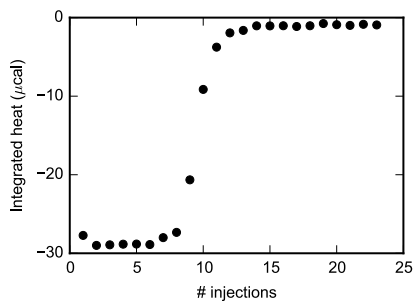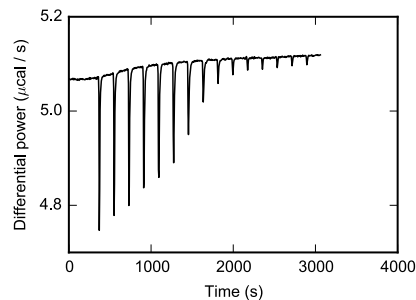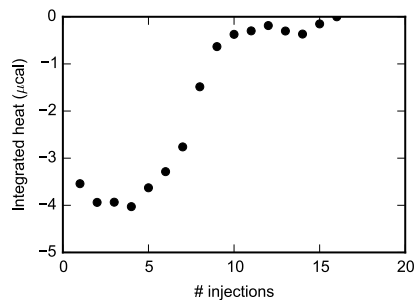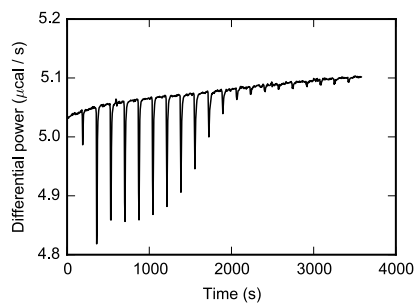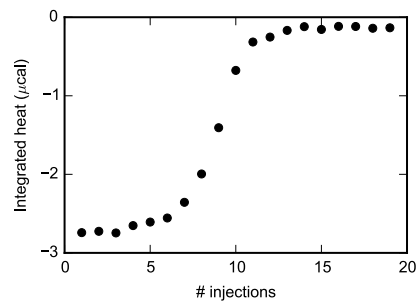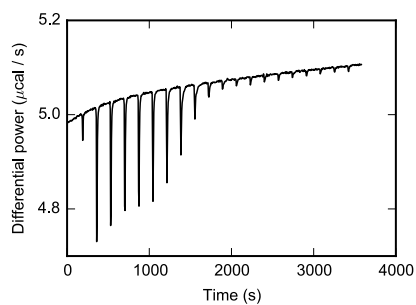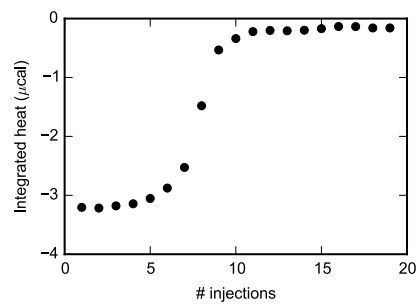

Supplement: S1 Fig — From top to bottom: Mg(II):EDTA, ligand 1:thermolysin, ligand 2:thermolysin and ligand 3:thermolysin. (PDF) [file pone.0203224.s003.pdf]

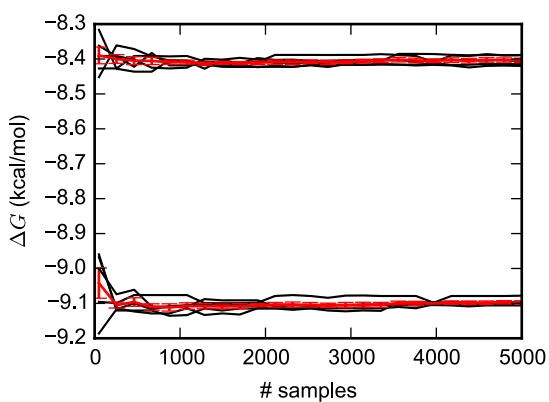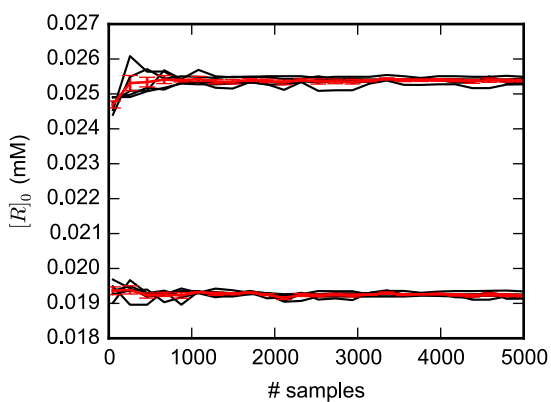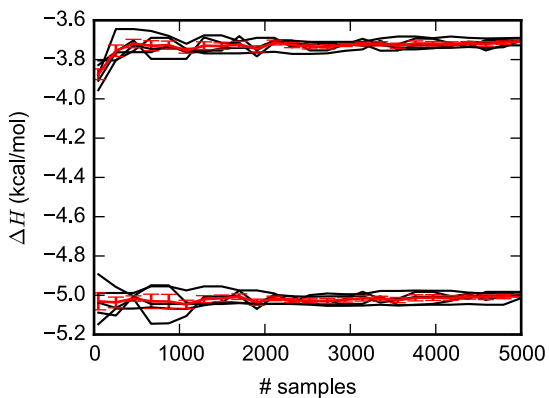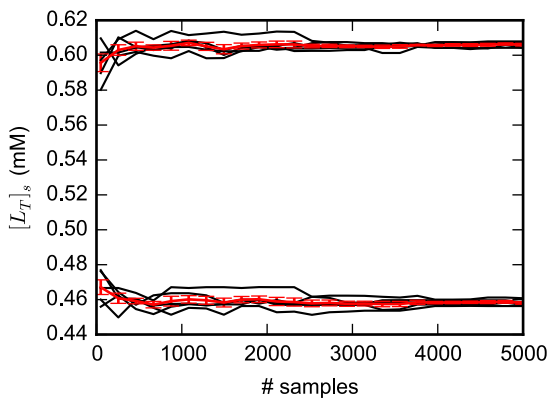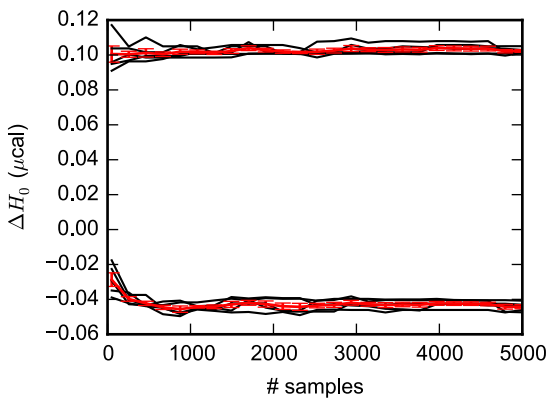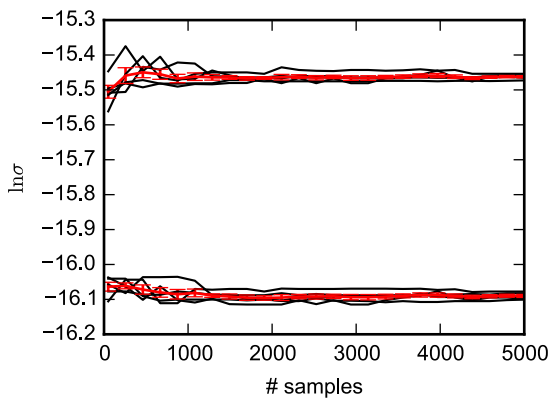

Supplement: S2 Fig — 5000 MCMC samples were generated from the Bayesian posterior (General model) for several variables based on one ITC dataset. For five independent repetitions of the MC simulations, the black lines are running estimates, as the number of samples is increased, of the upper and lower limits of 95% BCIs. The red line and error bars are the average and standard deviation across the five independent simulations. (PDF) [file pone.0203224.s004.pdf]

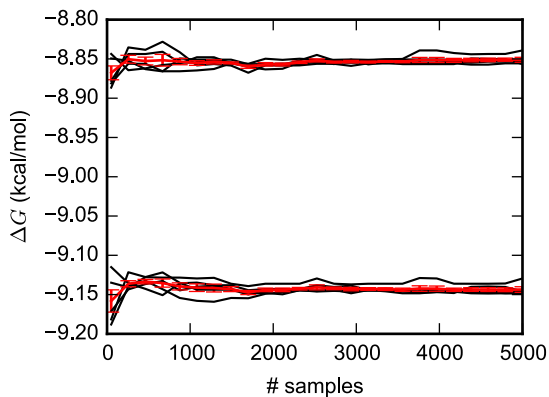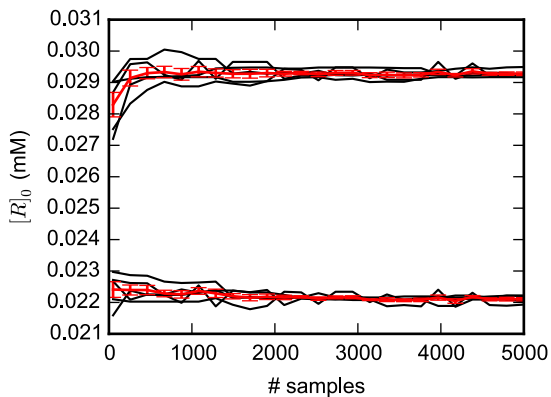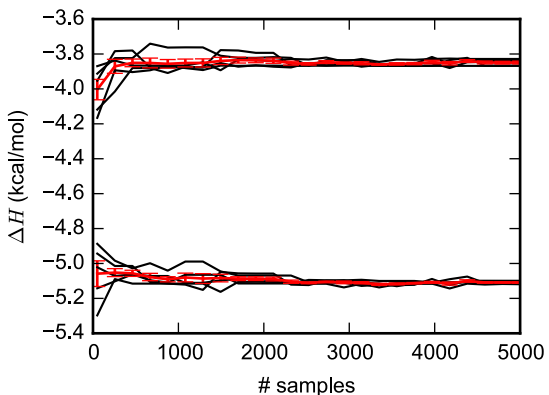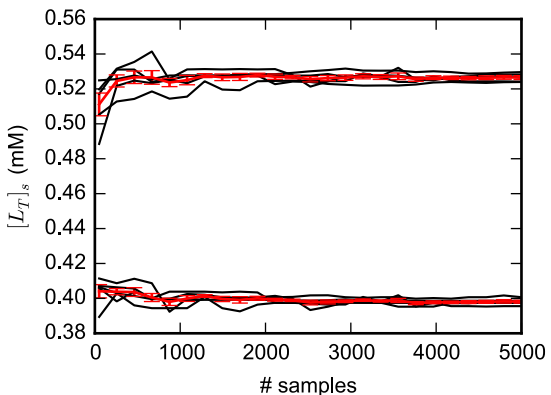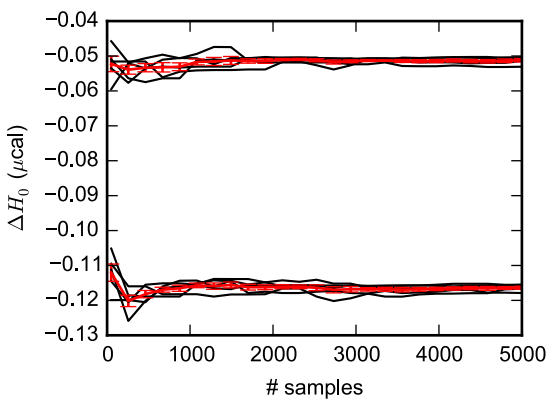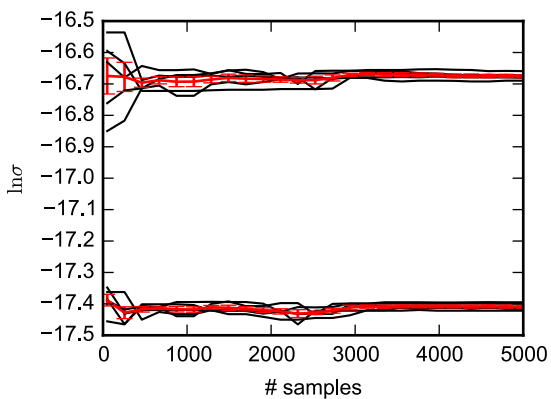

Supplement: S3 Fig — 5000 MCMC samples were generated from the Bayesian posterior (General model) for several variables based on one ITC dataset. For five independent repetitions of the MC simulations, the black lines are running estimates, as the number of samples is increased, of the upper and lower limits of 95% BCIs. The red line and error bars are the average and standard deviation across the five independent simulations. (PDF) [file pone.0203224.s005.pdf]

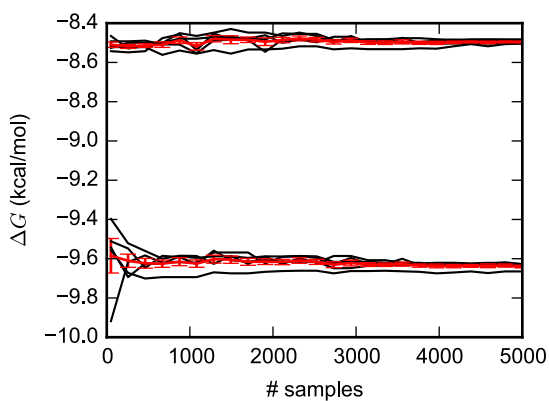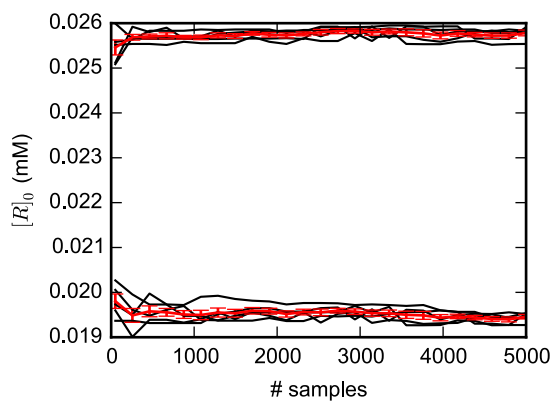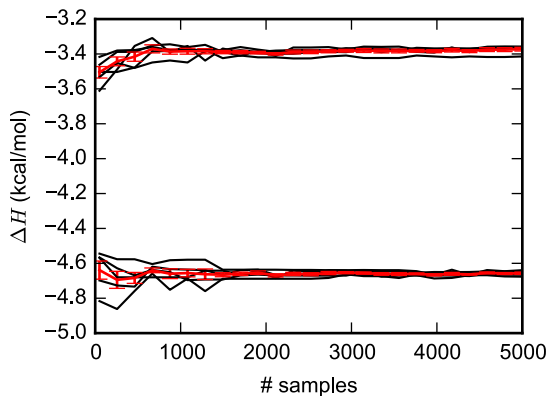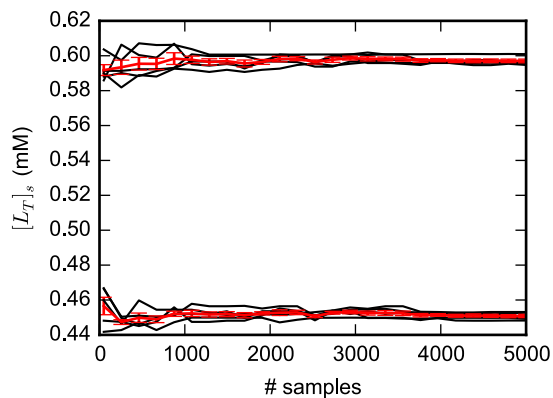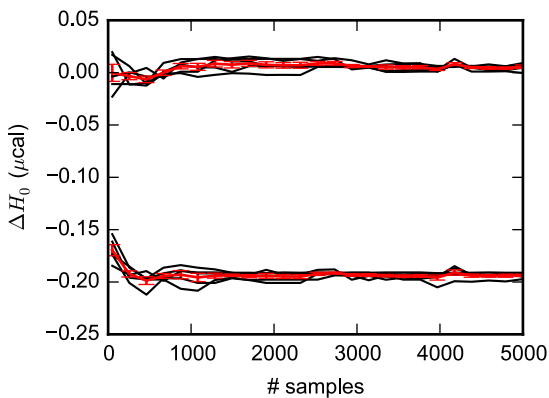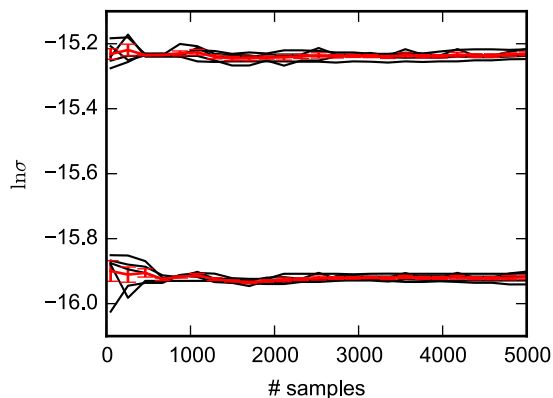

Supplement: S4 Fig — 5000 MCMC samples were generated from the Bayesian posterior (General model) for several variables based on one ITC dataset. For five independent repetitions of the MC simulations, the black lines are running estimates, as the number of samples is increased, of the upper and lower limits of 95% BCIs. The red line and error bars are the average and standard deviation across the five independent simulations. (PDF) [file pone.0203224.s006.pdf]

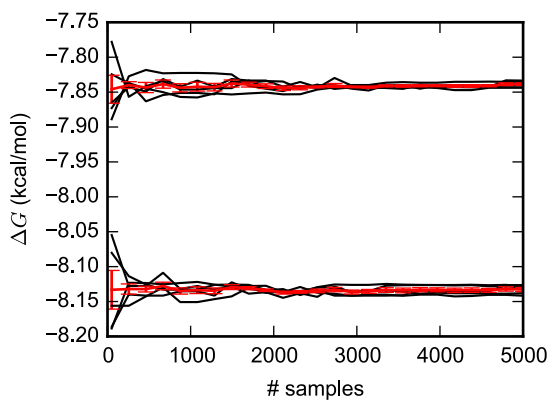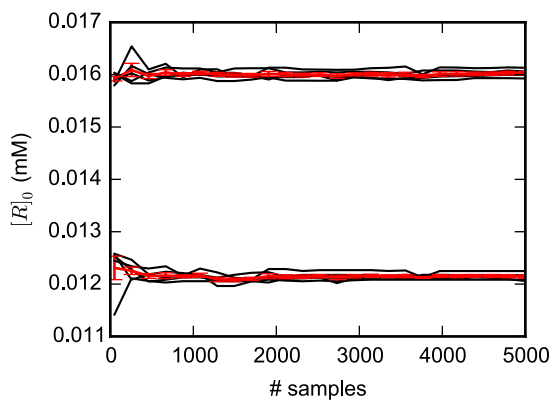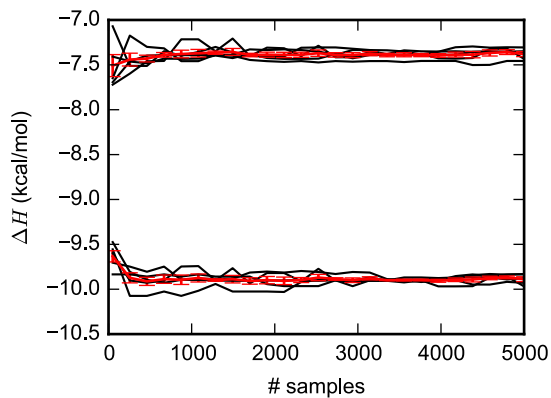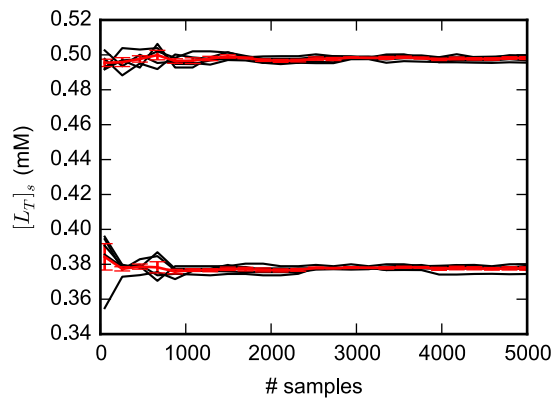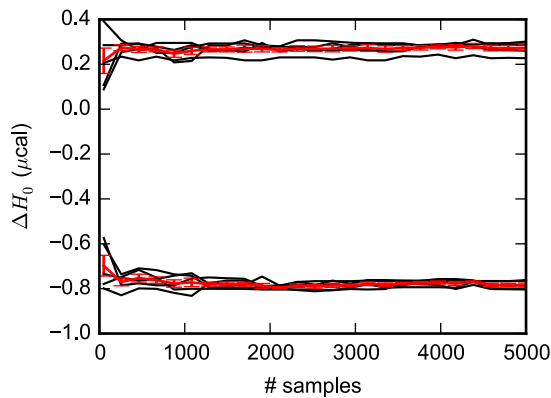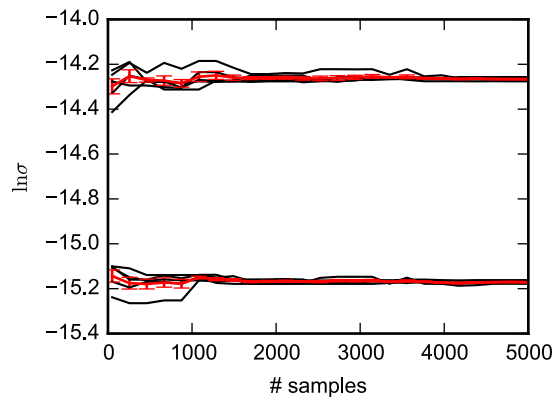

Supplement: S5 Fig — 5000 MCMC samples were generated from the Bayesian posterior (General model) for several variables based on one ITC dataset for binding of CBS to CAII digitized from the ABRF MIRG’02 paper [23]. For five independent repetitions of the MC simulations, the black lines are running estimates, as the number of samples is increased, of the upper and lower limits of 95% BCIs. The red line and error bars are the average and standard deviation across the five independent simulations. (PDF) [file pone.0203224.s007.pdf]

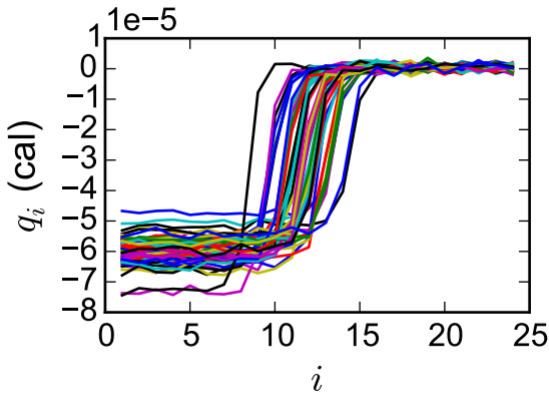

Supplement: S6 Fig — Parameters for the curves are in the Experimental section of the main text. (PDF) [file pone.0203224.s008.pdf]

## Bayesian approach

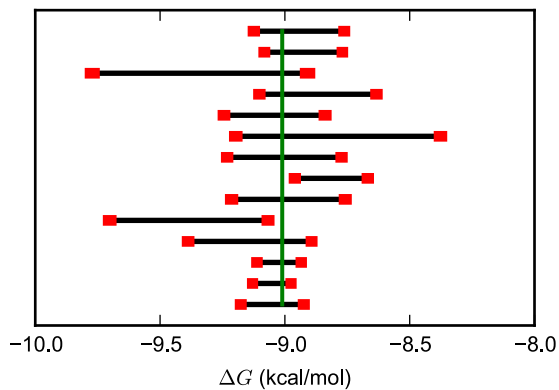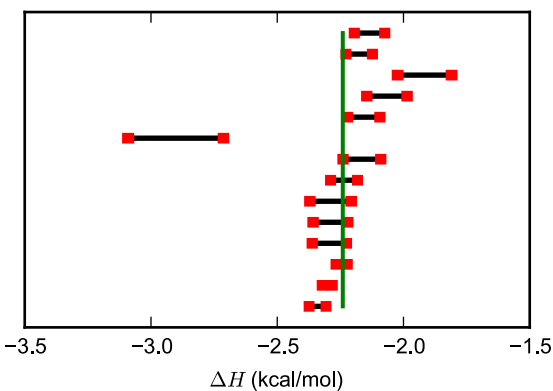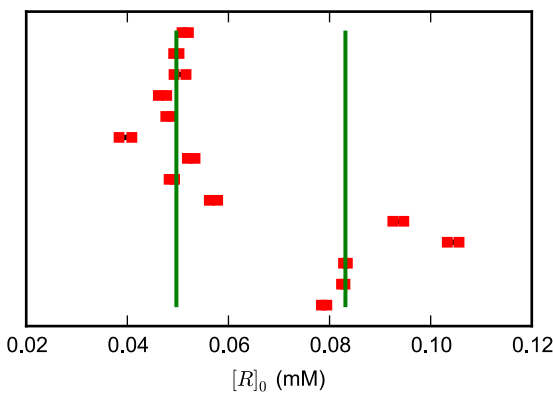

## Nonlinear least squares

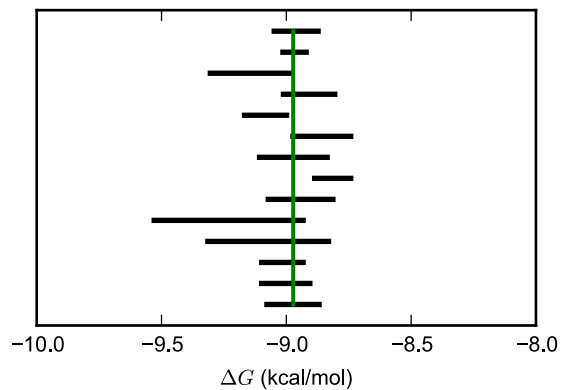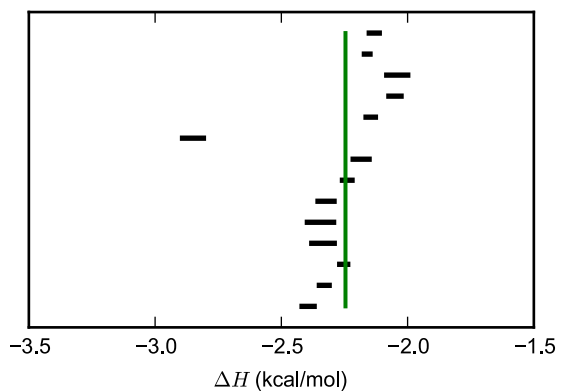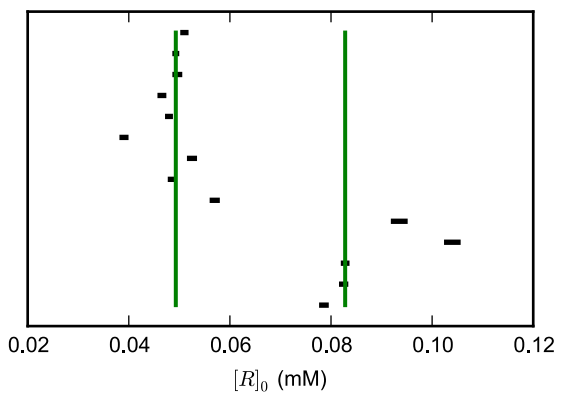

Supplement: S7 Fig — 95% credible intervals estimated from Bayesian analysis (left) and confidence intervals from nonlinear least squares (right) for parameters specifying magnesium binding to EDTA. The vertical green lines are the median. There are two median estimates for R because the experiments were done at two different concentrations. Red bars denote the standard deviations of the lower and upper bounds, estimated by bootstrapping, and are a total of two standard deviations wide. (PDF) [file pone.0203224.s009.pdf]

# Bayesian approach

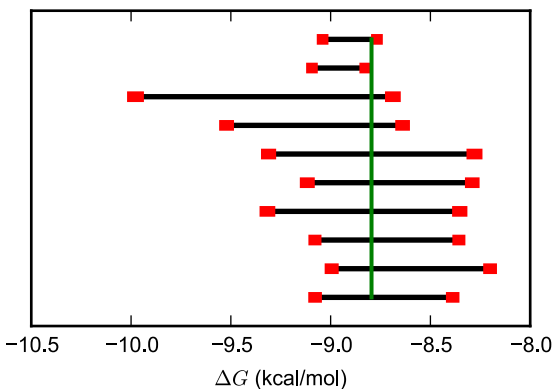

# Nonlinear least squares

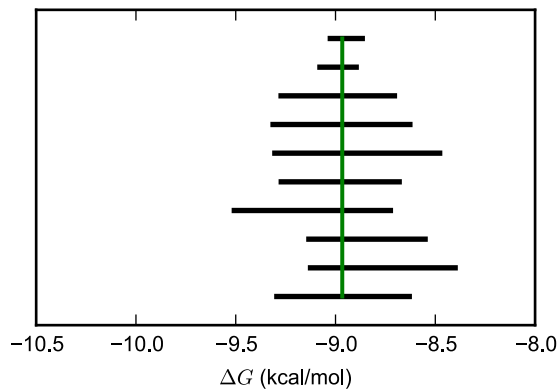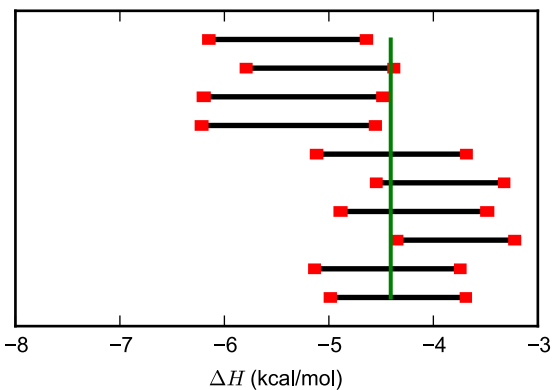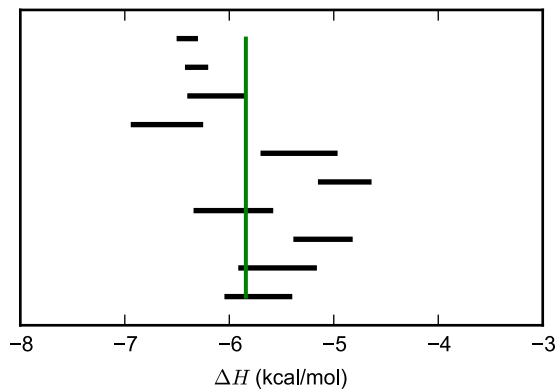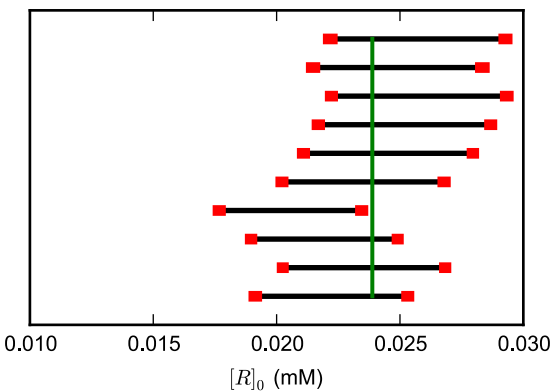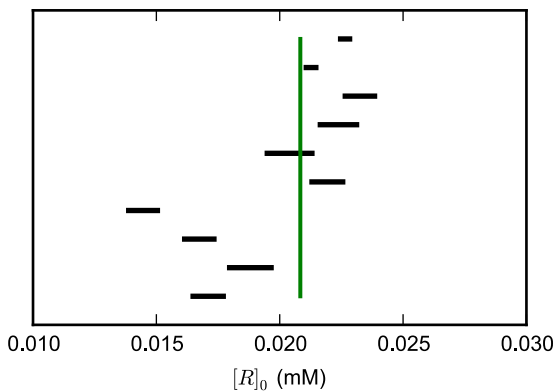

Supplement: S8 Fig — 95% credible intervals estimated from the Bayesian posterior (left) and confidence intervals from nonlinear least squares (right) for parameters specifying ligand 1 binding to thermolysin. The vertical green lines are the median. Red bars denote the standard deviations of the lower and upper bounds, estimated by bootstrapping, and are a total of two standard deviations wide. (PDF) [file pone.0203224.s010.pdf]

# Bayesian approach

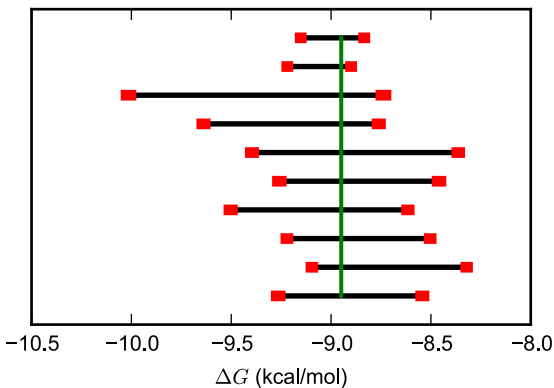

# Nonlinear least squares

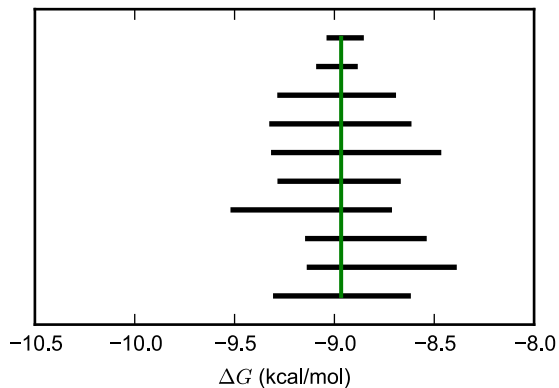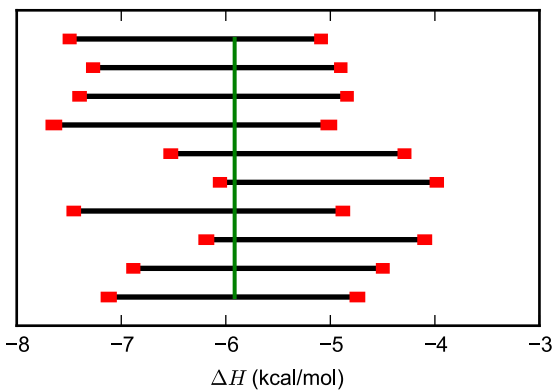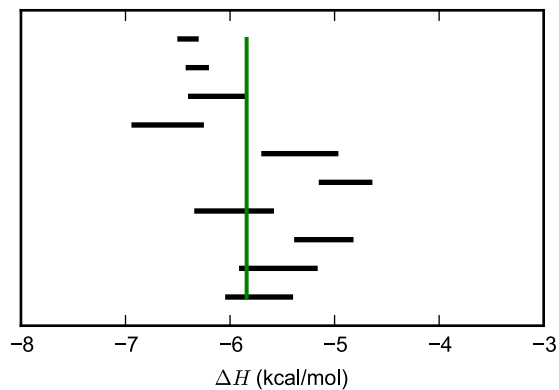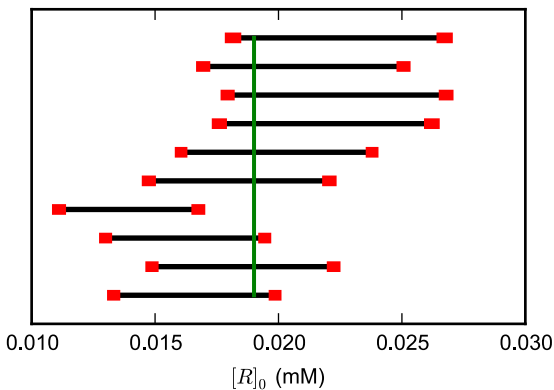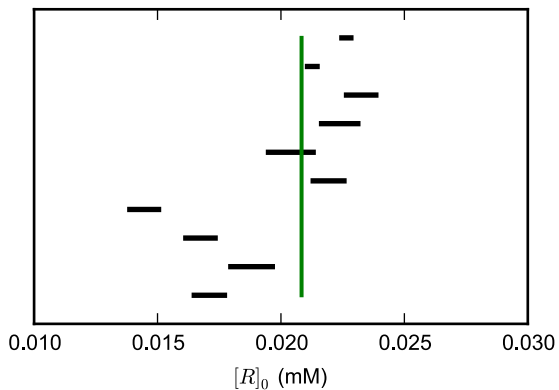

Supplement: S9 Fig — 95% credible intervals estimated from the Bayesian posterior (left) and confidence intervals from nonlinear least squares (right) for parameters specifying ligand 1 binding to thermolysin. The vertical green lines are the median. Red bars denote the standard deviations of the lower and upper bounds, estimated by bootstrapping, and are a total of two standard deviations wide. (PDF) [file pone.0203224.s011.pdf]

# Bayesian approach

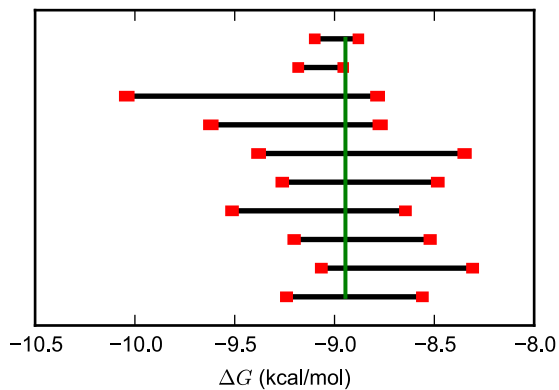

# Nonlinear least squares

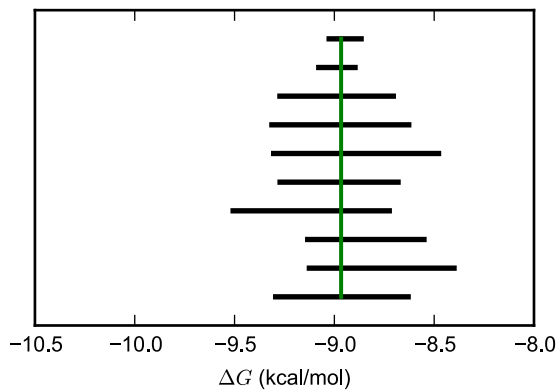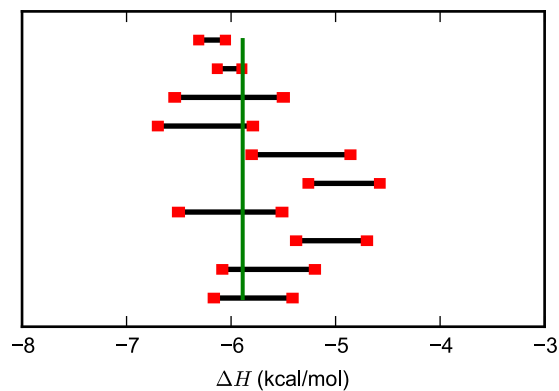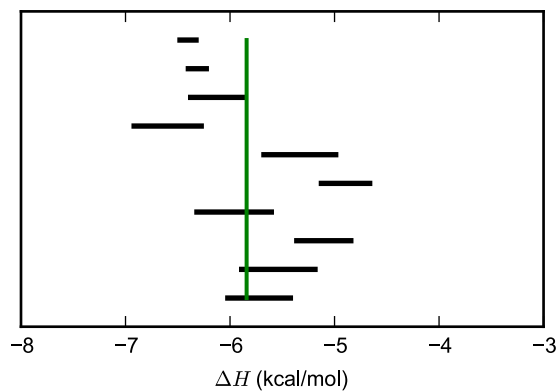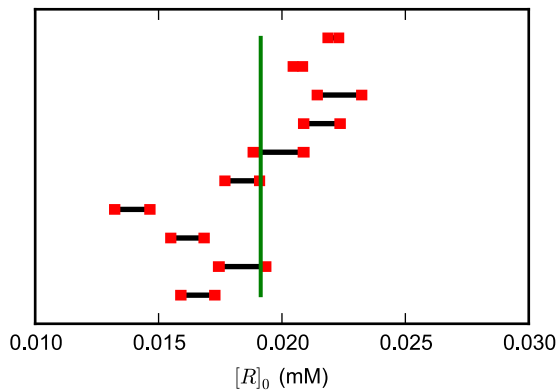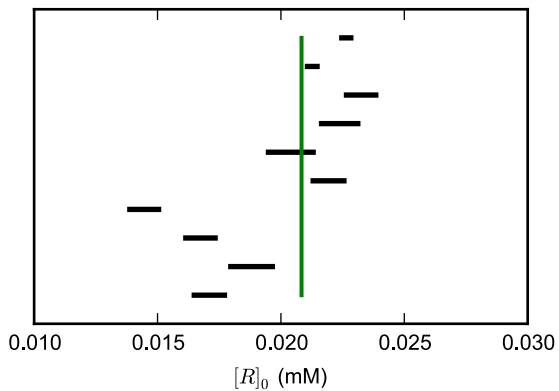

Supplement: S10 Fig — 95% credible intervals estimated from the Bayesian posterior (left) and confidence intervals from nonlinear least squares (right) for parameters specifying ligand 1 binding to thermolysin. The vertical green lines are the median. Red bars denote the standard deviations of the lower and upper bounds, estimated by bootstrapping, and are a total of two standard deviations wide. (PDF) [file pone.0203224.s012.pdf]

# Bayesian approach

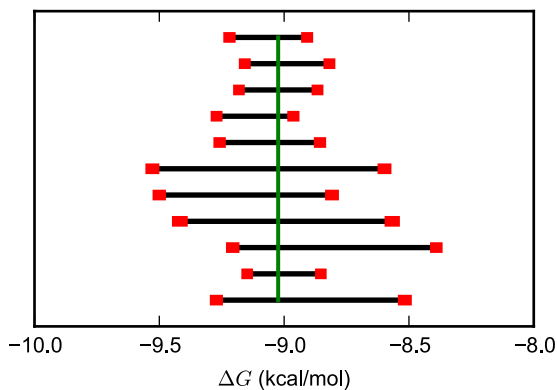

# Nonlinear least squares

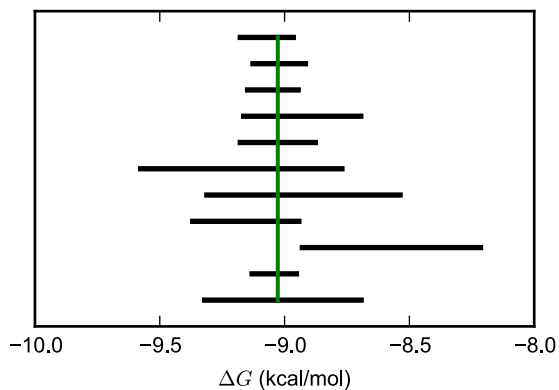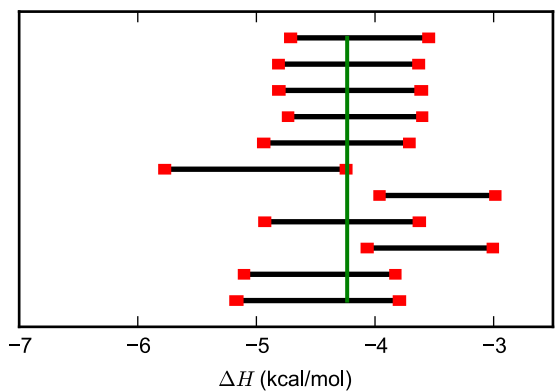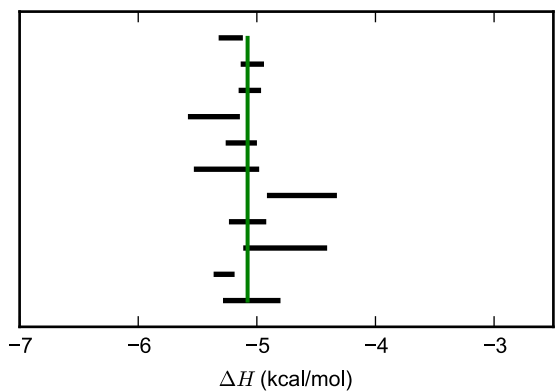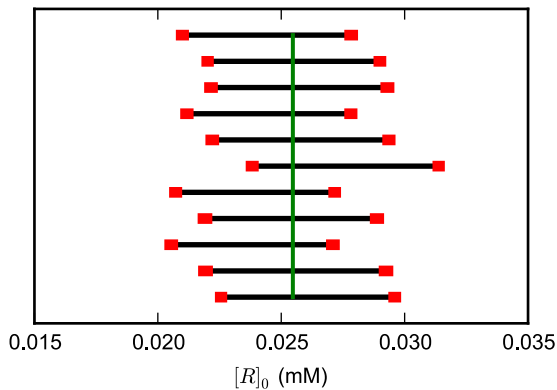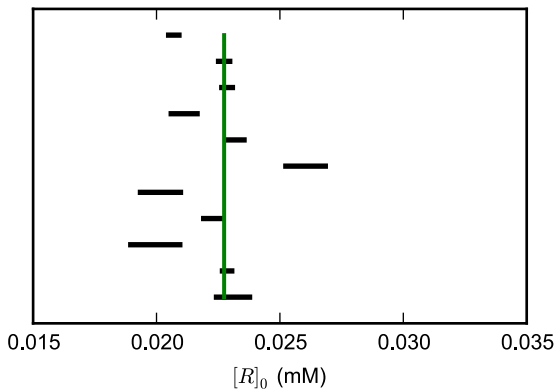

Supplement: S11 Fig — 95% credible intervals estimated from the Bayesian posterior (left) and confidence intervals from nonlinear least squares (right) for parameters specifying ligand 2 binding to thermolysin. The vertical green lines are the median. Red bars denote the standard deviations of the lower and upper bounds, estimated by bootstrapping, and are a total of two standard deviations wide. (PDF) [file pone.0203224.s013.pdf]

# Bayesian approach

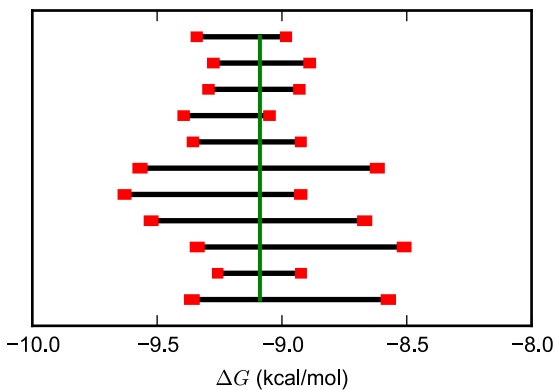

# Nonlinear least squares

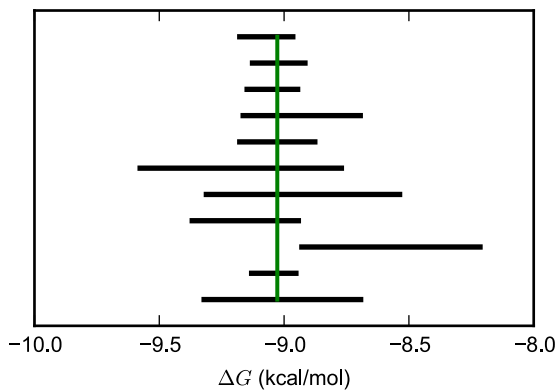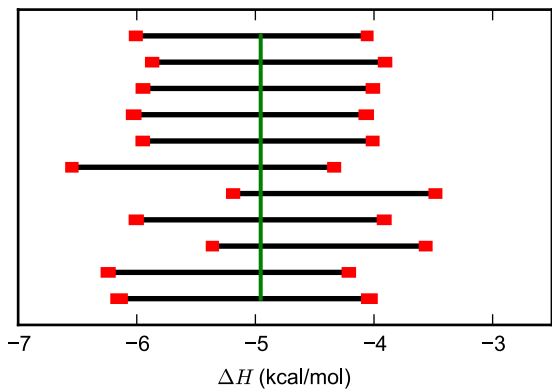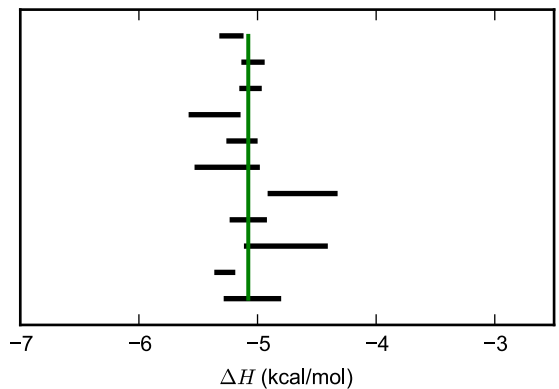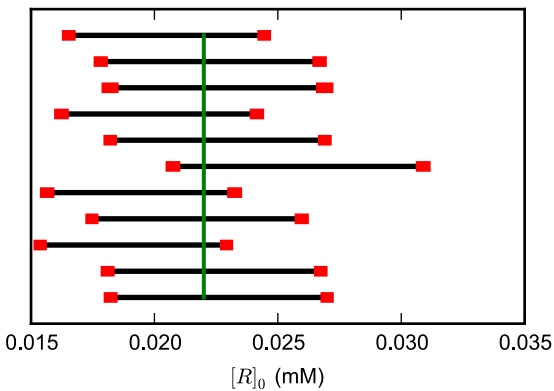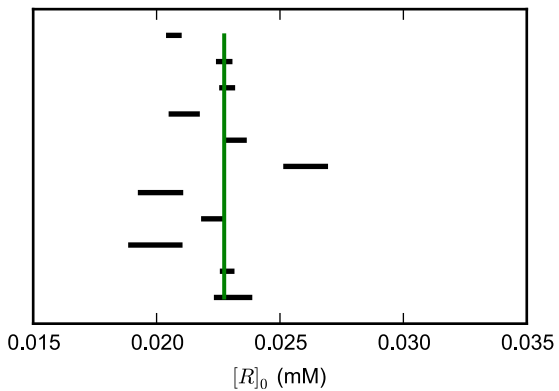

Supplement: S12 Fig — 95% credible intervals estimated from the Bayesian posterior (left) and confidence intervals from nonlinear least squares (right) for parameters specifying ligand 2 binding to thermolysin. The vertical green lines are the median. Red bars denote the standard deviations of the lower and upper bounds, estimated by bootstrapping, and are a total of two standard deviations wide. (PDF) [file pone.0203224.s014.pdf]

# Bayesian approach

# Nonlinear least squares

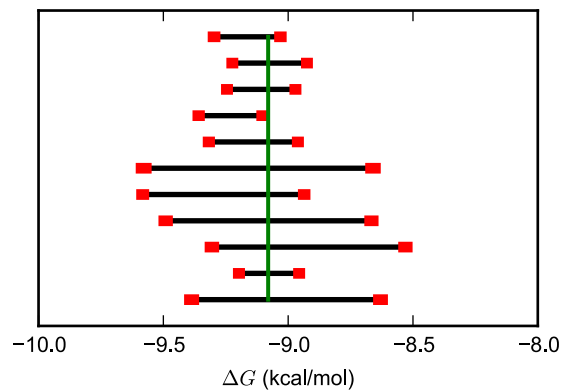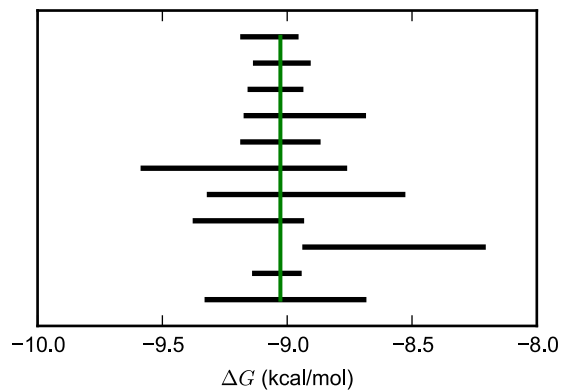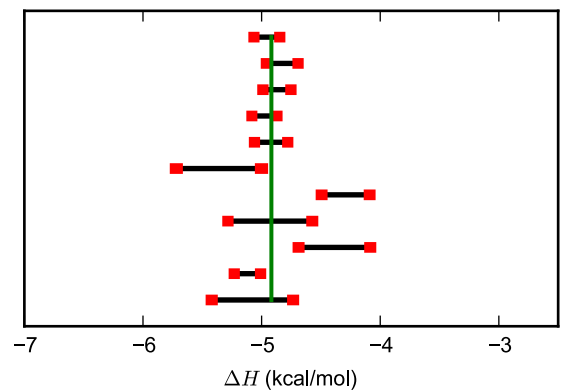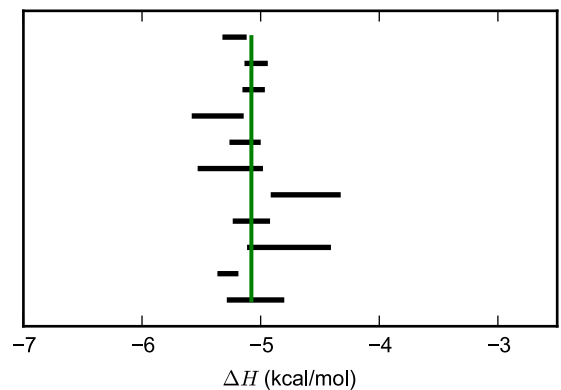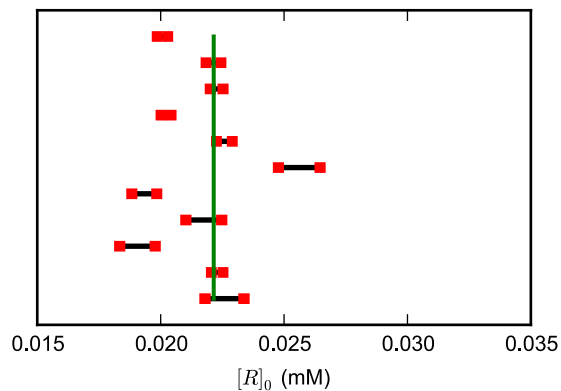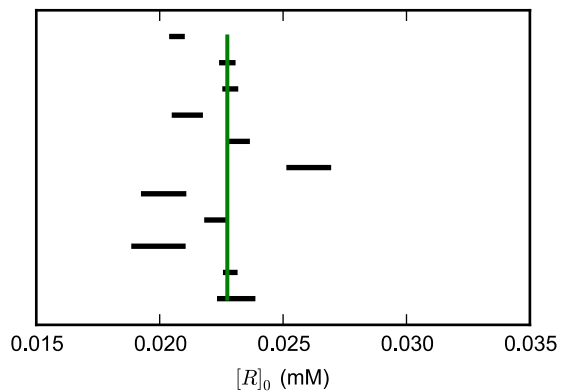

Supplement: S13 Fig — 95% credible intervals estimated from the Bayesian posterior (left) and confidence intervals from nonlinear least squares (right) for parameters specifying ligand 2 binding to thermolysin. The vertical green lines are the median. Red bars denote the standard deviations of the lower and upper bounds, estimated by bootstrapping, and are a total of two standard deviations wide. (PDF) [file pone.0203224.s015.pdf]

# Bayesian approach

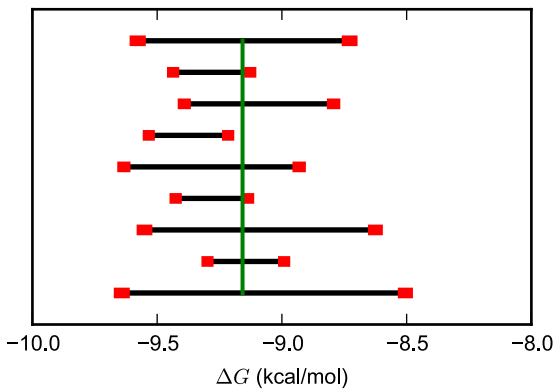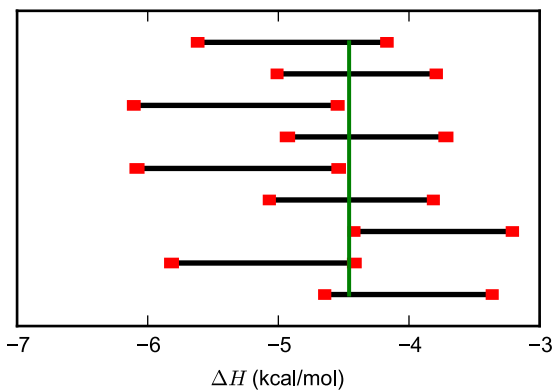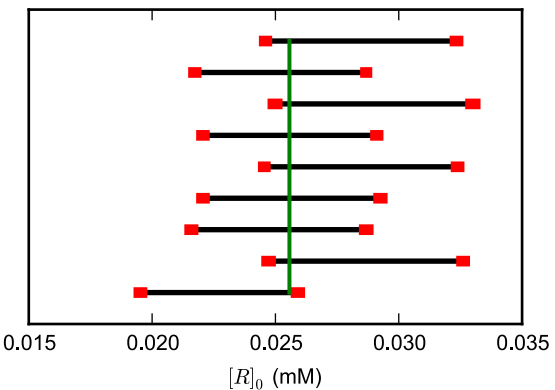

# Nonlinear least squares

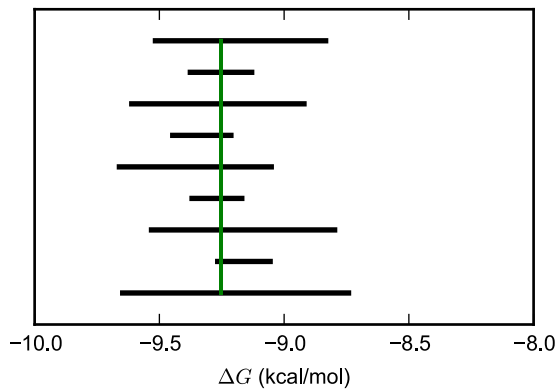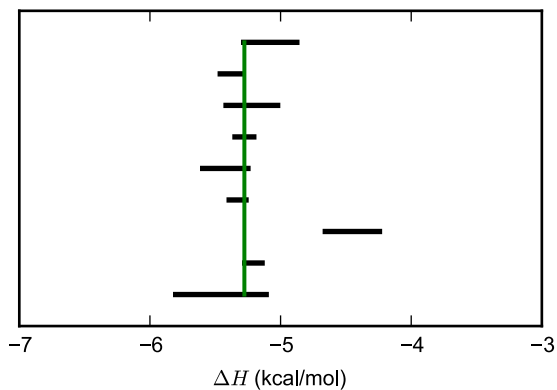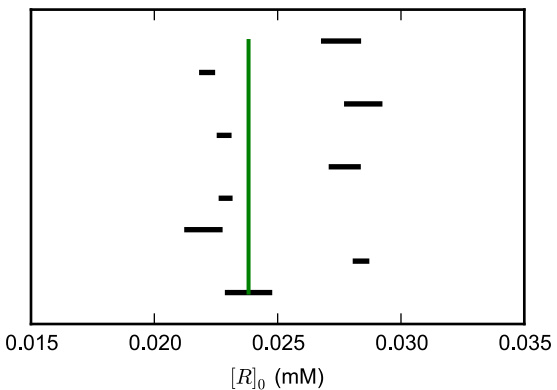

Supplement: S14 Fig — 95% credible intervals estimated from the Bayesian posterior (left) and confidence intervals from nonlinear least squares (right) for parameters specifying ligand 3 binding to thermolysin. The vertical green lines are the median. Red bars denote the standard deviations of the lower and upper bounds, estimated by bootstrapping, and are a total of two standard deviations wide. (PDF) [file pone.0203224.s016.pdf]

# Bayesian approach

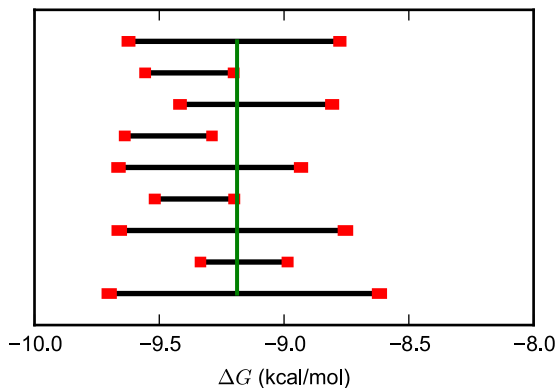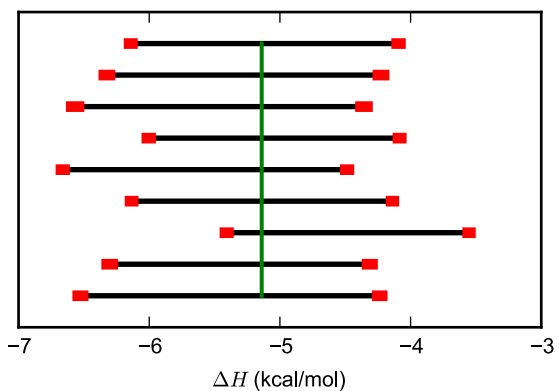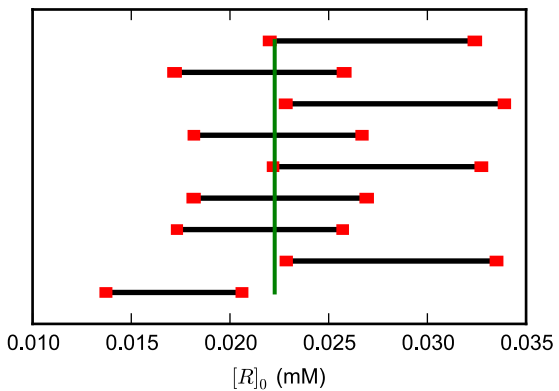

# Nonlinear least squares

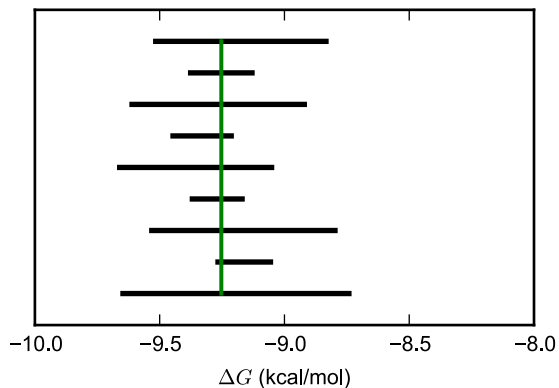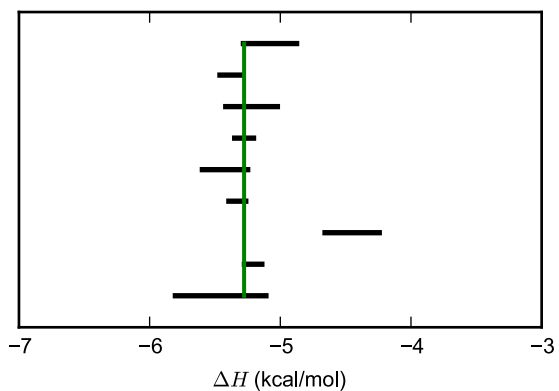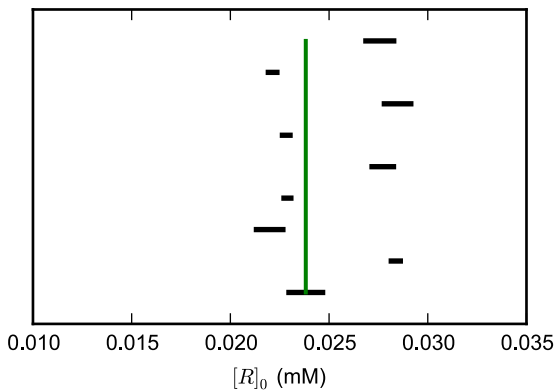

Supplement: S15 Fig — 95% credible intervals estimated from the Bayesian posterior (left) and confidence intervals from nonlinear least squares (right) for parameters specifying ligand 3 binding to thermolysin. The vertical green lines are the median. Red bars denote the standard deviations of the lower and upper bounds, estimated by bootstrapping, and are a total of two standard deviations wide. (PDF) [file pone.0203224.s017.pdf]

# Bayesian approach

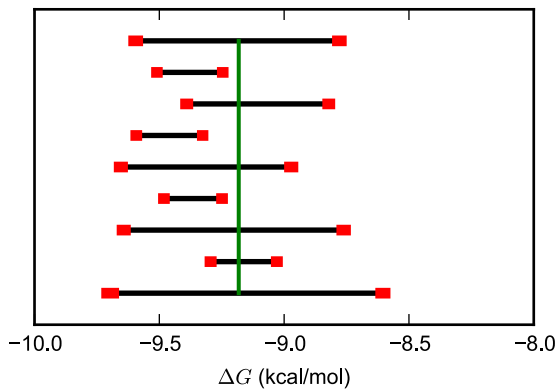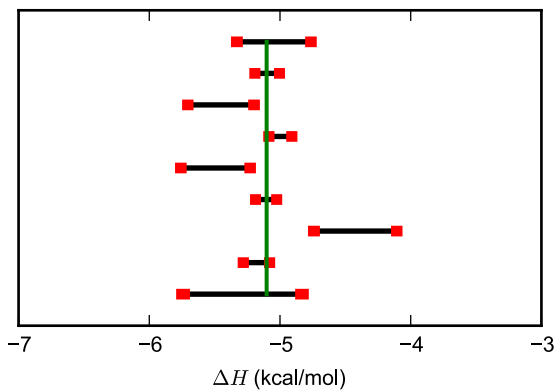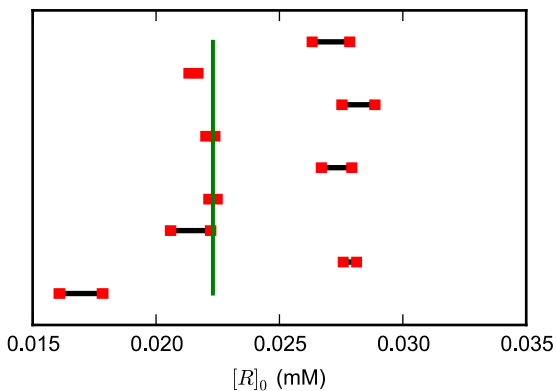

# Nonlinear least squares

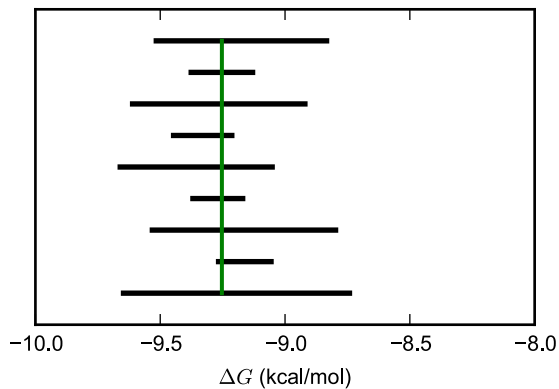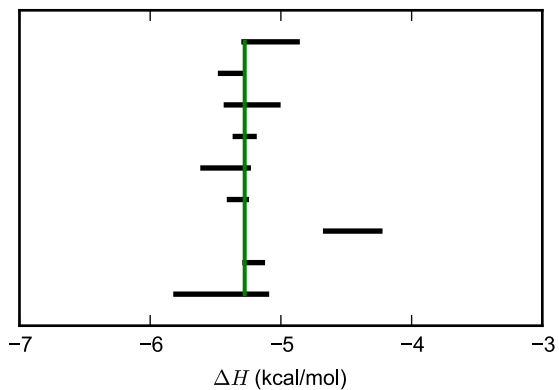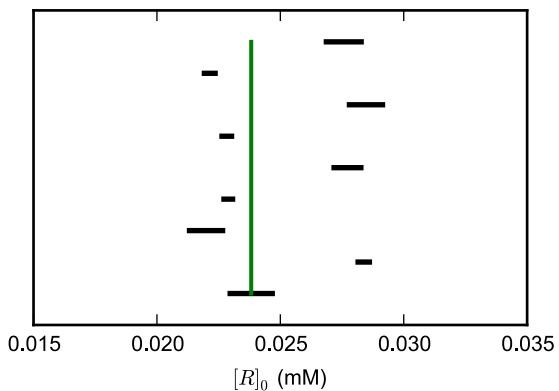

Supplement: S16 Fig — 95% credible intervals estimated from the Bayesian posterior (left) and confidence intervals from nonlinear least squares (right) for parameters specifying ligand 3 binding to thermolysin. The vertical green lines are the median. Red bars denote the standard deviations of the lower and upper bounds, estimated by bootstrapping, and are a total of two standard deviations wide. (PDF) [file pone.0203224.s018.pdf]

# Bayesian approach

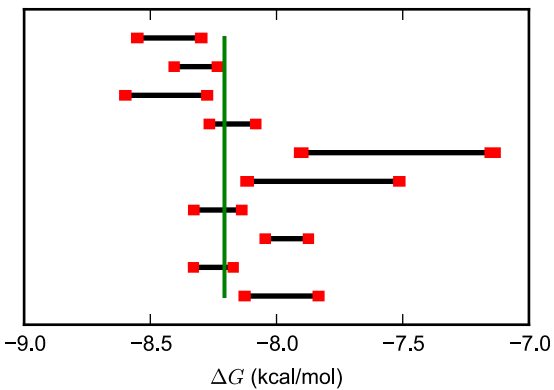

# Nonlinear least squares

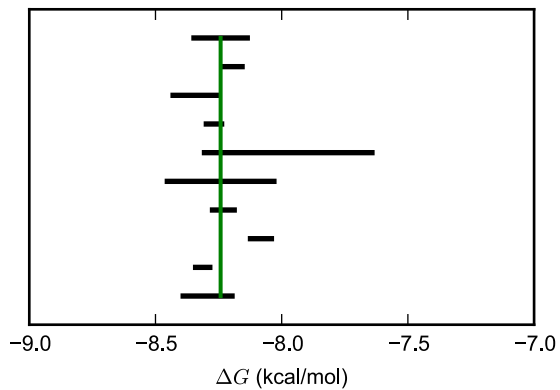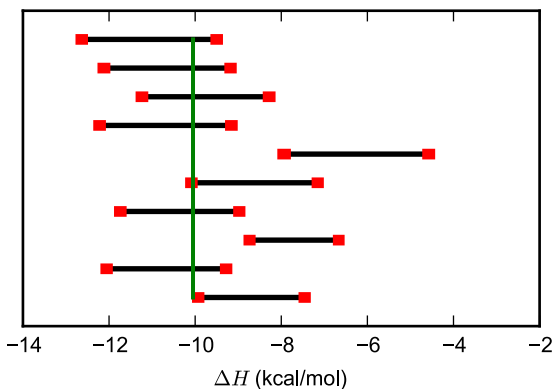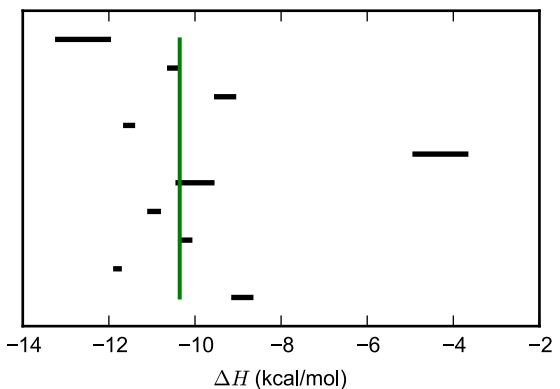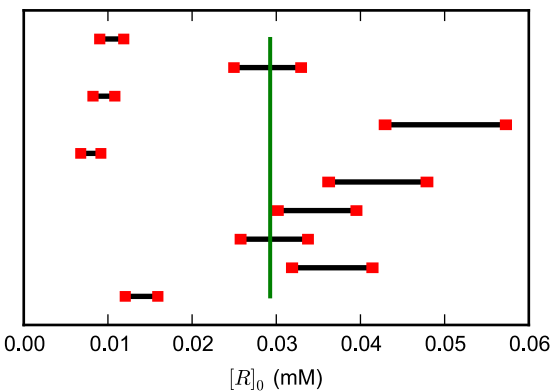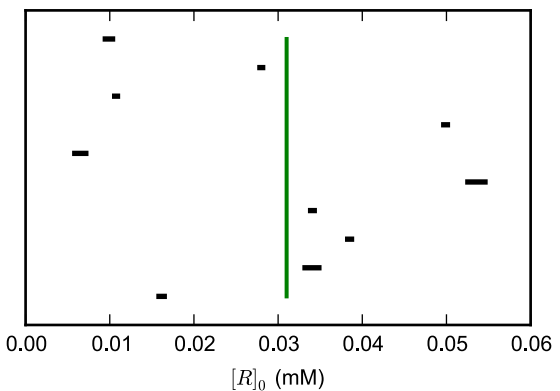

Supplement: S17 Fig — 95% credible intervals estimated from the Bayesian posterior (left) and confidence intervals from nonlinear least squares (right) for parameters specifying CBS binding to CAII. The vertical green lines are the median. Note that each experiment was done at different concentration. Red bars denote the standard deviations of the lower and upper bounds, estimated by bootstrapping, and are a total of two standard deviations wide. (PDF) [file pone.0203224.s019.pdf]

# Bayesian approach

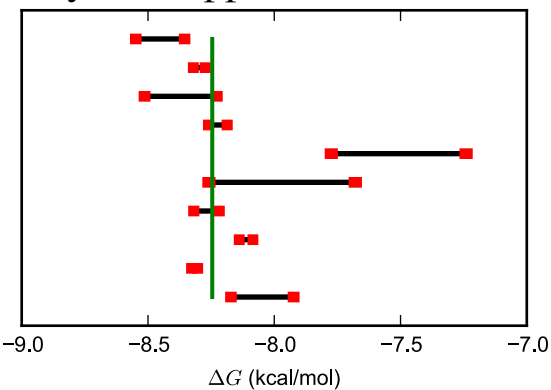

# Nonlinear least squares

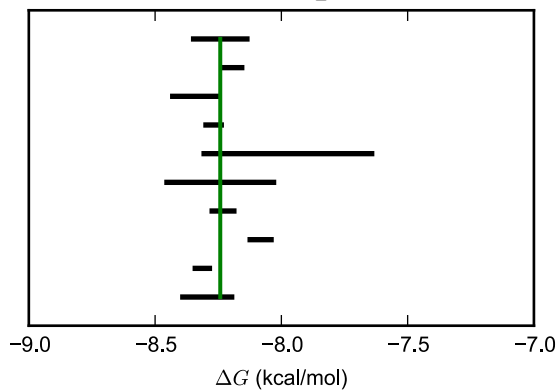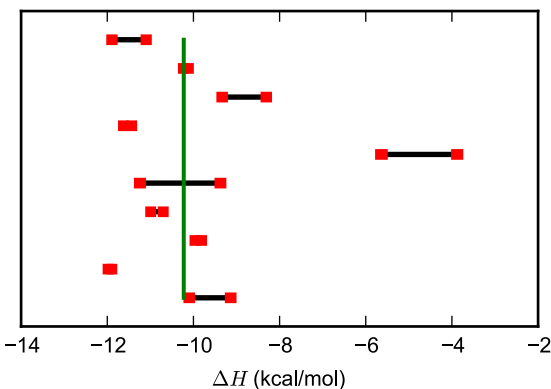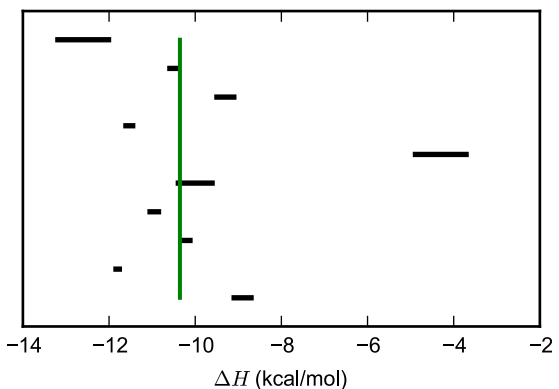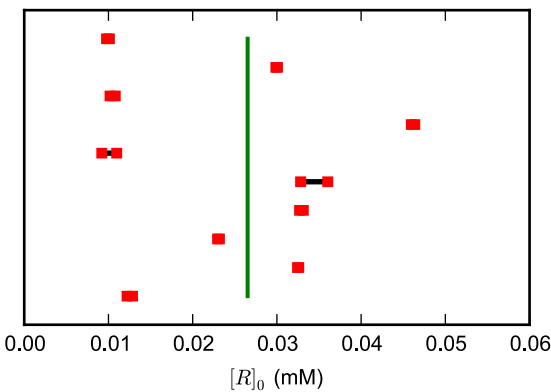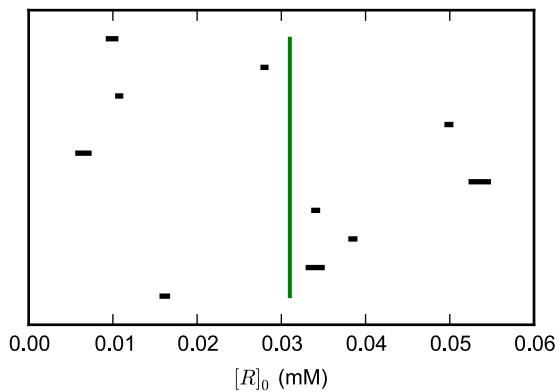

Supplement: S18 Fig — 95% credible intervals estimated from the Bayesian analysis (left) and confidence intervals from nonlinear least squares (right) for parameters specifying CBS binding to CAII. The vertical green lines are the median. Note that each experiment was done at different concentration. Red bars denote the standard deviations of the lower and upper bounds, estimated by bootstrapping, and are a total of two standard deviations wide. (PDF) [file pone.0203224.s020.pdf]

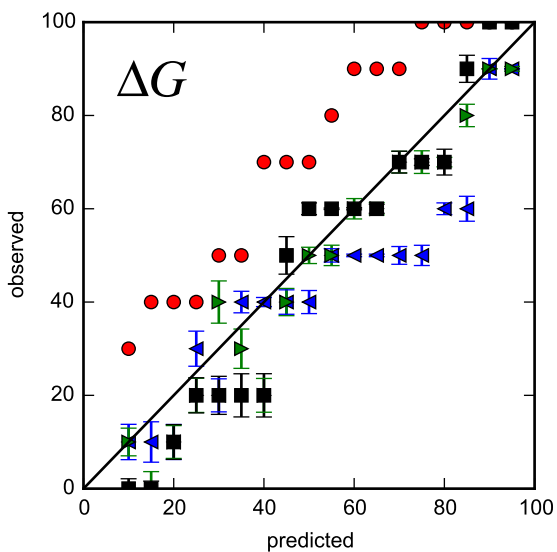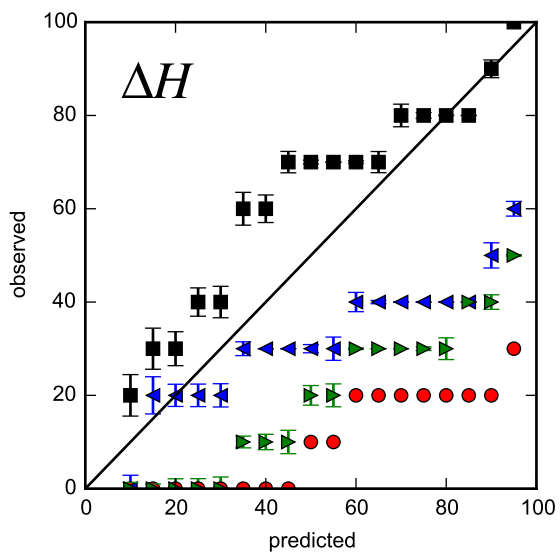

Supplement: S19 Fig — For the ligand 1:thermolysin experiments, the predicted versus observed rate (%) in which intervals contain the median value for binding parameters is shown. Intervals were BCIs based on the General (blue leftward triangles), Flat [R]0 (black squares), and Comparison (green rightward triangles) models or nonlinear least squares confidence intervals (red circles). Error bars are standard deviations based on bootstrapping. (PDF) [file pone.0203224.s021.pdf]

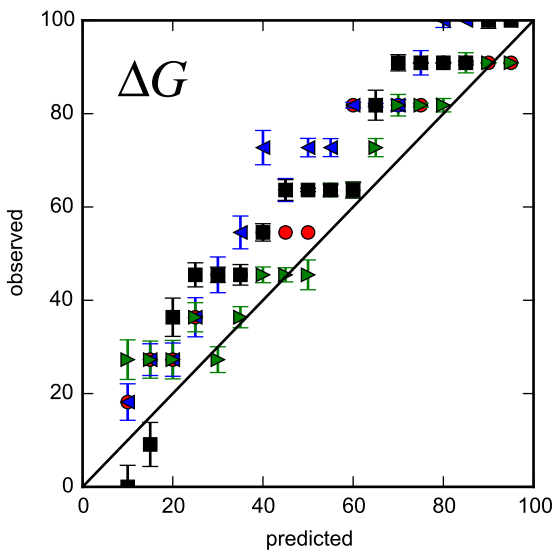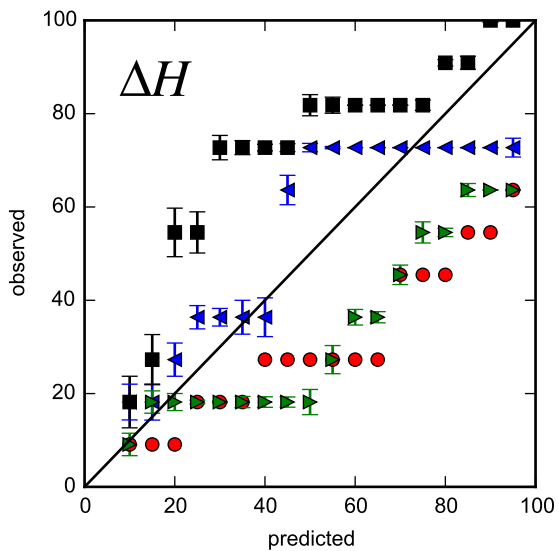

Supplement: S20 Fig — For the ligand 2:thermolysin experiments, the predicted versus observed rate (%) in which intervals contain the median value for binding parameters is shown. Intervals were BCIs based on the General (blue leftward triangles), Flat [R]0 (black squares), and Comparison (green rightward triangles) models or nonlinear least squares confidence intervals (red circles). Error bars are standard deviations based on bootstrapping. (PDF) [file pone.0203224.s022.pdf]

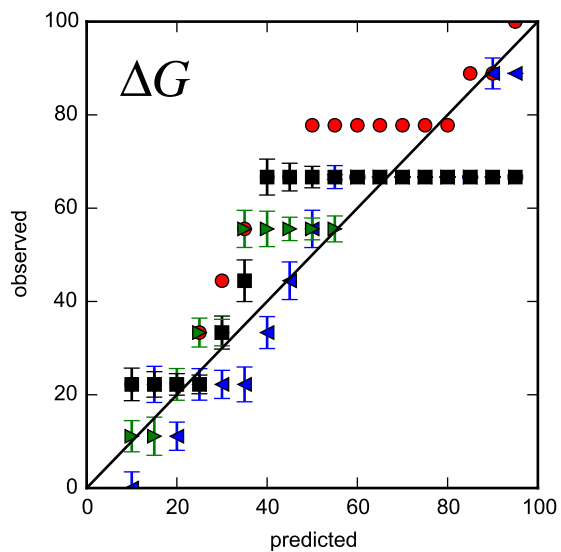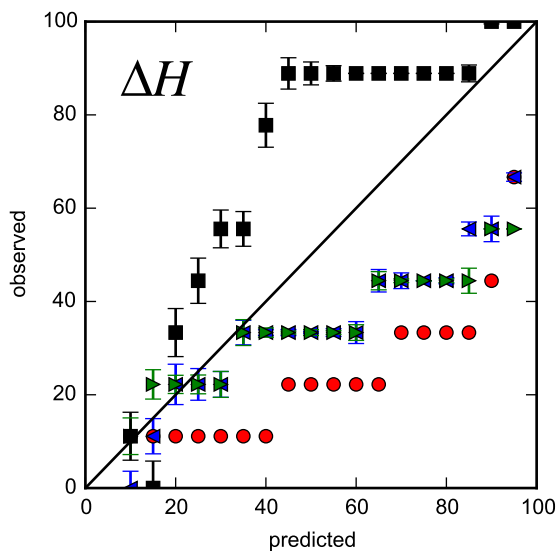

Supplement: S21 Fig — For the ligand 3:thermolysin experiments, the predicted versus observed rate (%) in which intervals contain the median value for binding parameters is shown. Intervals were BCIs based on the General (blue leftward triangles), Flat [R]0 (black squares), and Comparison (green rightward triangles) models or nonlinear least squares confidence intervals (red circles). Error bars are standard deviations based on bootstrapping. (PDF) [file pone.0203224.s023.pdf]

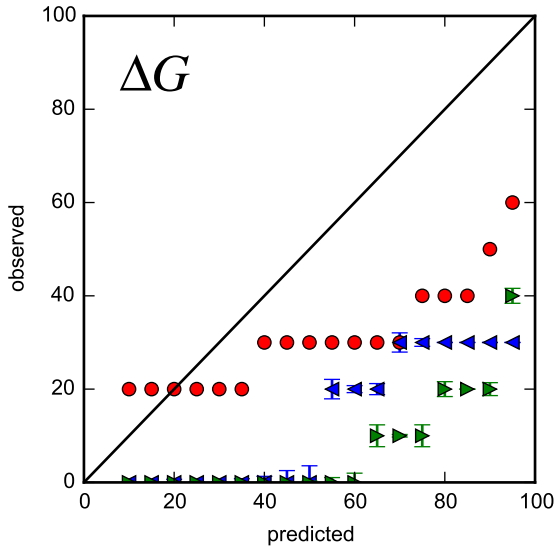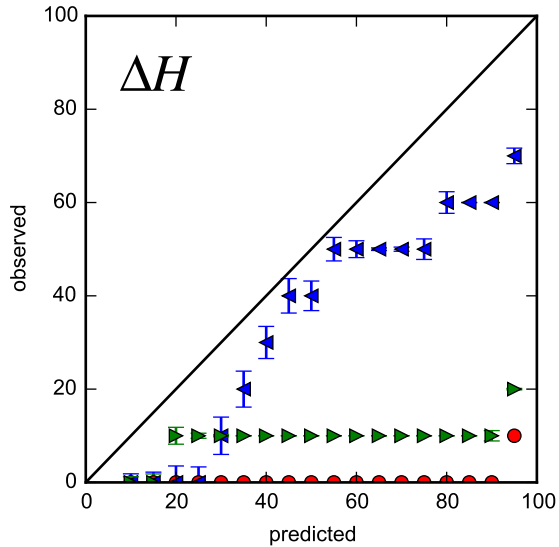

Supplement: S22 Fig — For the CBS:CAII experiments, the predicted versus observed rate (%) in which intervals contain the median value for binding parameters is shown. Intervals were BCIs based on the General (blue leftward triangles), Flat [R]0 (black squares), and Comparison (green rightward triangles) models or nonlinear least squares confidence intervals (red circles). Error bars are standard deviations based on bootstrapping. (PDF) [file pone.0203224.s024.pdf]

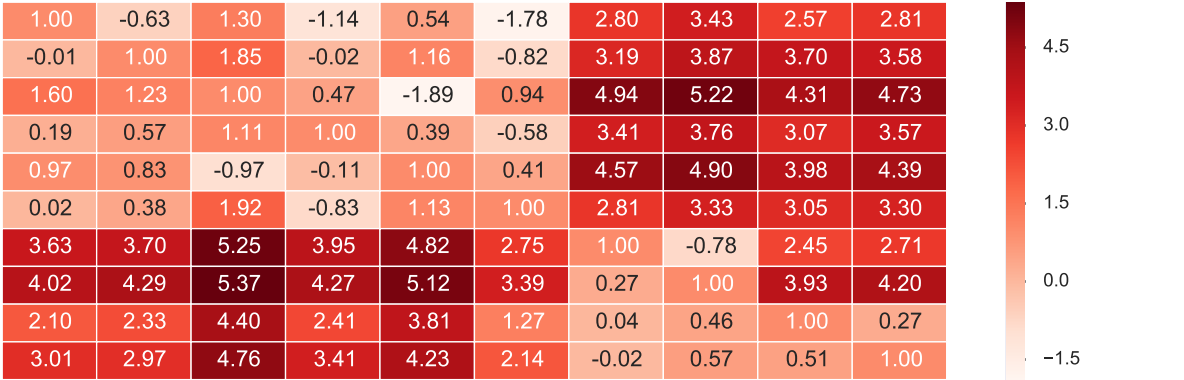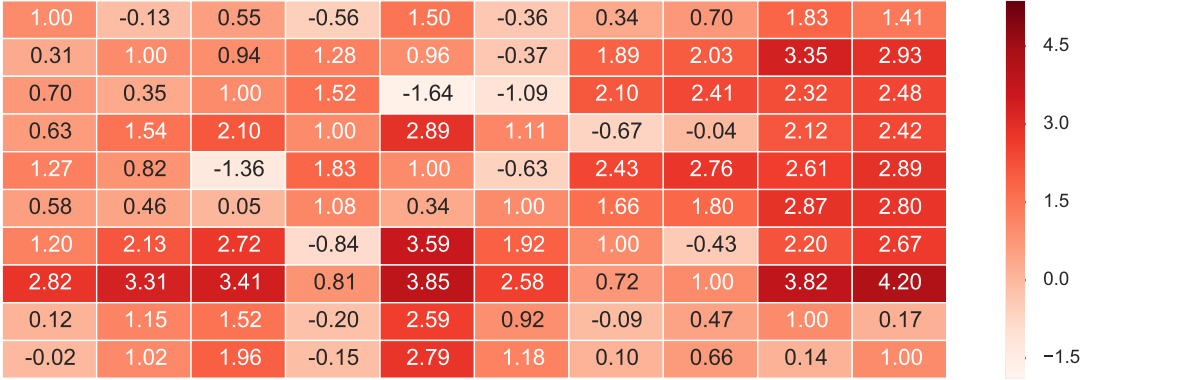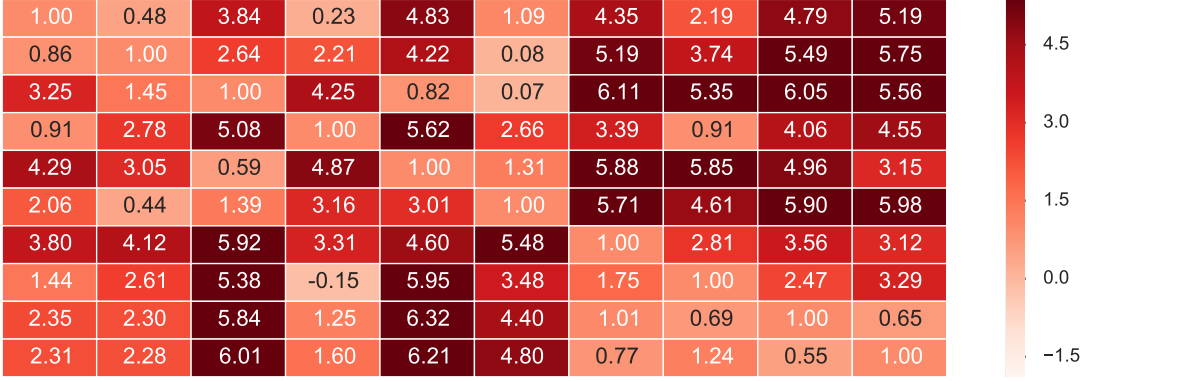

Supplement: S23 Fig — Each column and row corresponds to one of the 10 datasets of ligand 1:thermolysin binding. The diagonal elements should be ln0 = −∞ but were set to 1 for visualization. (PDF) [file pone.0203224.s025.pdf]

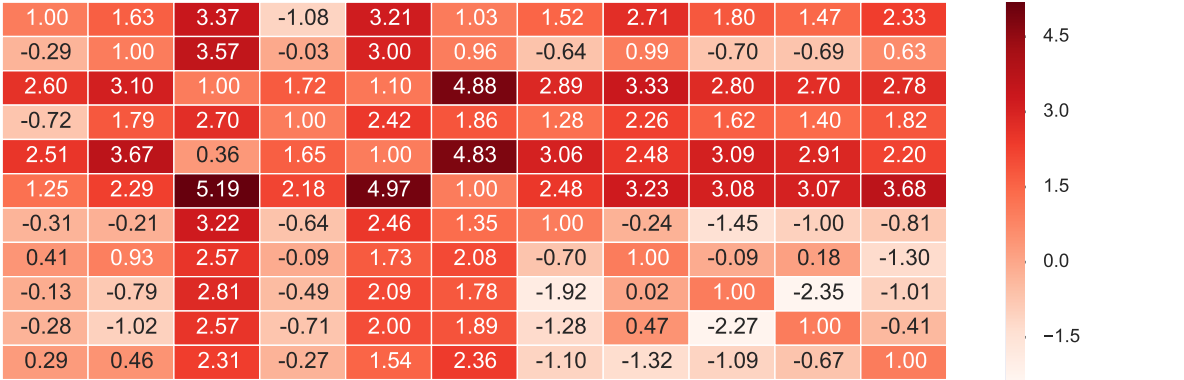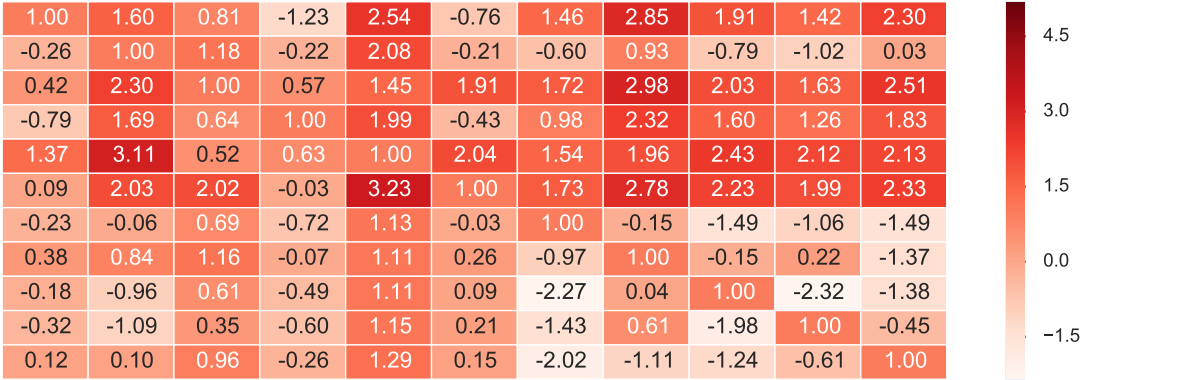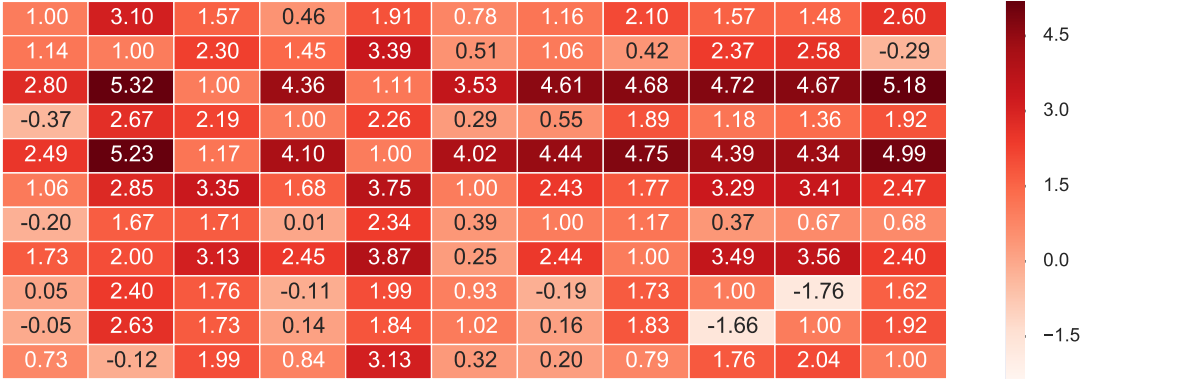

Supplement: S24 Fig — Each column and row corresponds to one of the 11 datasets of ligand 2:thermolysin binding. The diagonal elements should be ln0 = −∞ but were set to 1 for visualization. (PDF) [file pone.0203224.s026.pdf]

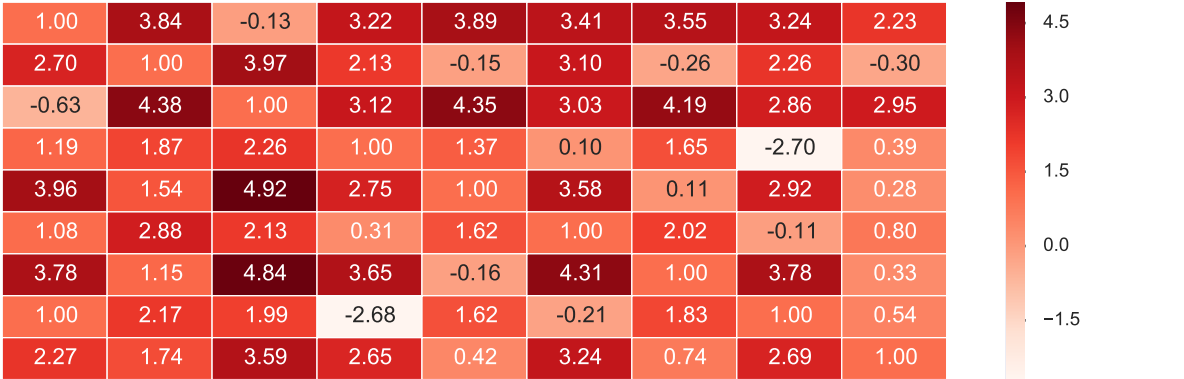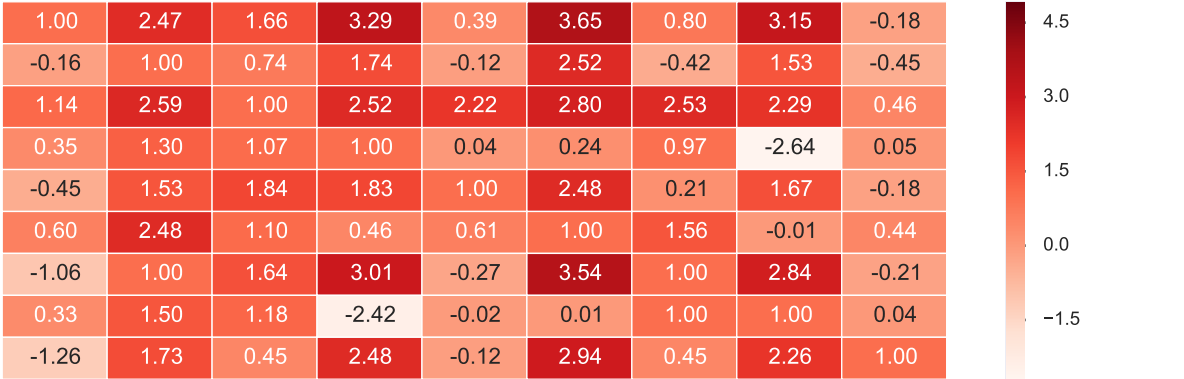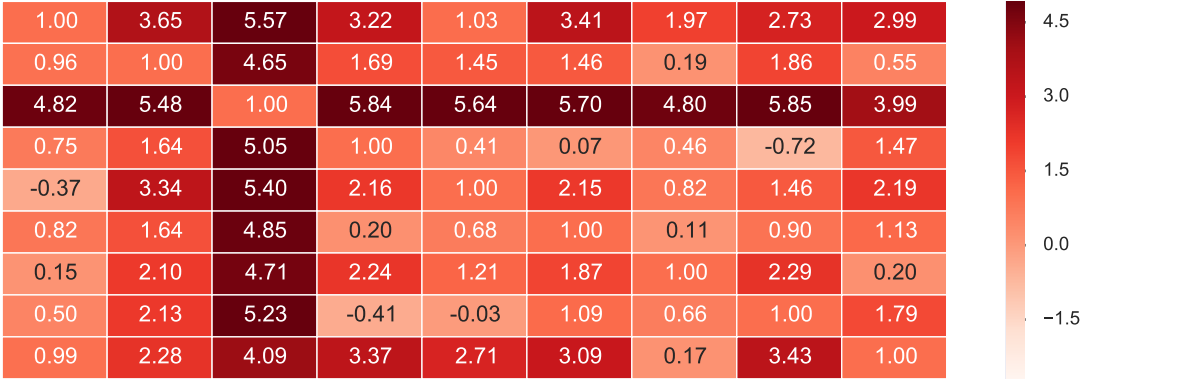

Supplement: S25 Fig — Each column and row corresponds to one of the 11 datasets of ligand 3:thermolysin binding. The diagonal elements should be ln0 = −∞ but were set to 1 for visualization. (PDF) [file pone.0203224.s027.pdf]

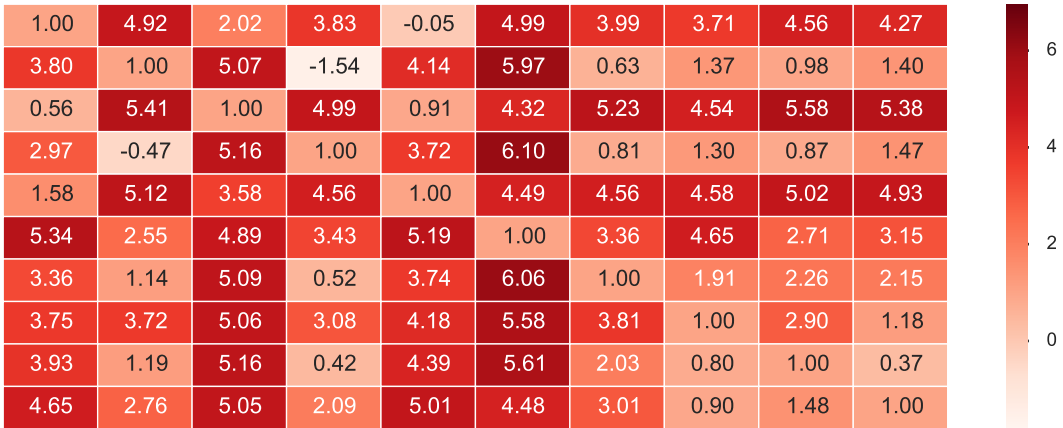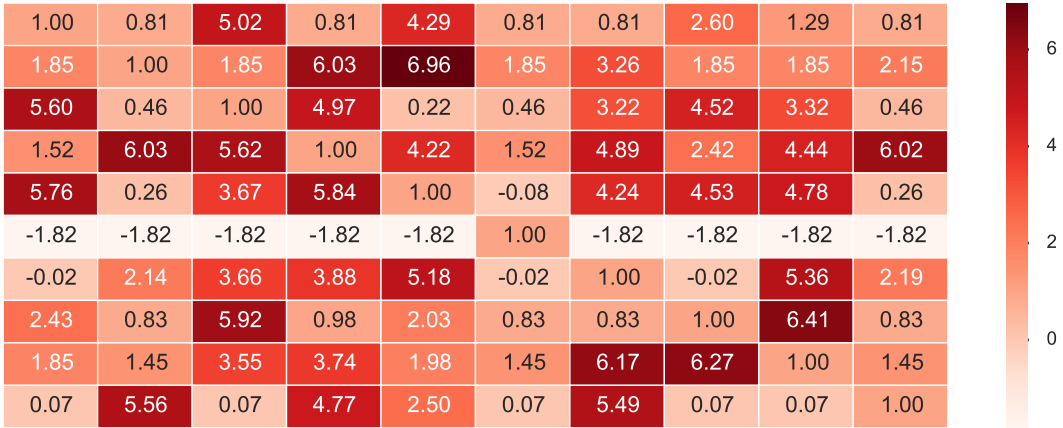

Supplement: S26 Fig — Each column and row corresponds to one of the 10 datasets of CBS:CAII binding. The diagonal elements should be ln0 = −∞ but were set to 1 for visualization. (PDF) [file pone.0203224.s028.pdf]
